# Anode Free Zinc‐Metal Batteries (AFZMBs): A New Paradigm in Energy Storage

**DOI:** 10.1002/smll.202412161

**Published:** 2025-03-04

**Authors:** Sunny Nandi, Martin Pumera

**Affiliations:** ^1^ New Technologies–Research Centre University of West Bohemia Univerzitní 8 Plzeň 30614 Czech Republic; ^2^ Faculty of Electrical Engineering and Computer Science VSB–Technical University of Ostrava 17. listopadu 2172/15 Ostrava 70800 Czech Republic

**Keywords:** 3D printing, advantages and challenges, anode free zinc‐metal batteries, current collector, electrolyte optimization, strategies

## Abstract

In the past few years, aqueous zinc‐metal batteries (ZMBs) have gained much attention and can be regarded as a potential alternative to lithium‐metal batteries owing to their high safety, nature of abundance, and environmental sustainability. However, several challenges persist, including dendrite formation, corrosion, and unwanted side reactions, before ZMBs can be fully utilized in practical applications. To circumvent these issues, anode free zinc‐metal batteries (AFZMBs) have emerged as a next‐generation energy storage system. This review provides a comprehensive analysis of recent developments in AFZMBs, including their working mechanisms, advantages over conventional ZMBs, and the challenges for practical implementation. It also highlights the key strategies, including current collector modification, electrolyte engineering, and 3D printing techniques to enhance zinc deposition uniformity and cycling stability. The review also explores how 3D printing technology can revolutionize the design of advanced current collectors and zinc‐rich cathodes, optimizing material utilization and enhancing battery performance. Finally, with a future perspective of AFZMBs is concluded, highlighting the need for further research to address existing bottlenecks and fully unlock their potential for next‐generation energy storage.

## Graphical abstract

Anode‐free zinc‐metal batteries (AFZMBs) are attracting considerable interest due to their high safety and energy density. This review explores the recent advances in AFZMBs, including their origin, working principles, advantages, challenges, and key strategies. It also spotlights the role of 3D printing techniques in large‐scale applications and provides insights into the future development of AFZMBs.

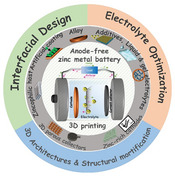

## 1 Introduction

**Figure 1:**
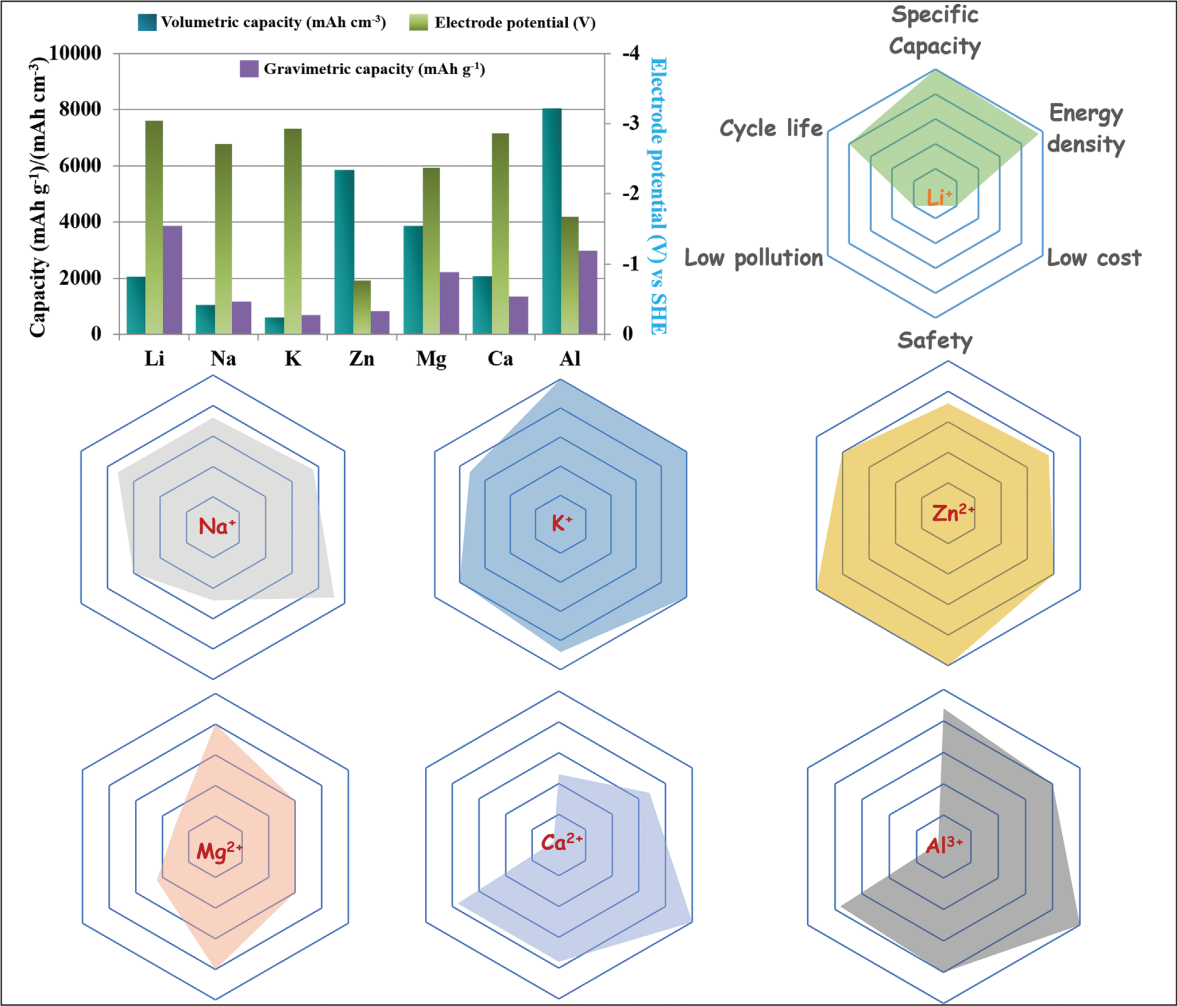
Comparison of lithium, sodium, potassium, zinc, magnesium, calcium, and aluminum based on volumetric capacity, gravimetric capacity, and electrode potential, as well as their respective radar plots.^[^ ^10^ ^]^

The rapid expansion of energy storage applications necessitates the development of high‐energy‐density and cost‐effective solutions to tackle the ongoing environmental and energy crisis.^[^ ^1^, ^2^ ^]^ Lithium‐ion batteries (LIBs) presently dominate the global energy market due to their certain attributes, including high energy density, long cycle life, high power density, etc.^[^ ^3^, ^4^, ^5^ ^]^ These attributes have made LIBs the favored option for a wide range of applications, ranging from miniature electronic devices to electric vehicles.^[^ ^5^, ^6^ ^]^ However, despite their extensive use, LIBs confront several limitations, including the paucity of lithium supplies, escalating costs, and safety issues associated with thermal runaway.^[^ ^7^ ^]^ These limitations hinder significant challenges for scaling up LIB technology, particularly in the field of sustainable energy storage systems. Consequently, there has been an upsurge in interest in alternative battery chemistries based on aqueous electrolytes that rely on abundant elements such as sodium, zinc, calcium, aluminum, etc., as shown in **Figure** **1**, and their values are tabulated in **Table** **1**.^[^ ^8^, ^9^, ^10^, ^11^, ^12^, ^13^, ^14^ ^]^

**Table 1:** Comparison and characteristics of charge carries with special focus on Li^+^ and Zn^2+^ ions.

Charge Carriers 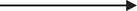	Li^+^	Na^+^	K^+^	Zn^2+^	Mg^2+^	Ca^2+^	Al^3+^
Resource ranking	33	6	7	25	8	5	3
Atomic weight (g mol^−1^)	6.94	22.99	39.1	65.41	24.31	40.38	26.98
Standard potential (V vs SHE)	−3.04	−2.713	−2.924	−0.763	−2.356	−2.84	−1.676
Ionic radius (Å)	0.76	1.02	1.38	0.75	0.72	1	0.53
Hydrated ionic radius (Å)	3.82	3.58	3.31	4.3	4.28	4.12	4.75
Volumetric energy density (mAh cm^−3^)	2061	1129	610	5855	3834	2072	8046
Specific capacity (mAh g^−1^)	3860	1166	685	820	2206	1337	2980
Metal cost (USD kg^−1^)	19.2	3.1	13.1	2.2	2.2	2.28	1.9
Density of the metal (kg m^−3^)	534	986	862	7140	1738	1550	2700

Among these alternatives, ZMBs have emerged as a promising candidate due to zinc's abundance, high safety, and environmental friendliness.^[^ ^14^, ^15^ ^]^ However, the current performance of ZMBs still falls short of meeting the requirements for practical applications. One of the key challenges lies in utilizing zinc (Zn) as an anode, particularly with the formation of dendrites during repeated battery cycling.^[^ ^16^, ^17^, ^18^, ^19^ ^]^ These dendrites can puncture the separator, leading to short circuits and significantly reducing the battery's lifespan. Additionally, the presence of a thick Zn metal anode, required for practical applications, not only increases the weight of the battery but also decreases the energy density.

To address these challenges, researchers have recently proposed anode‐free batteries.^[^ ^20^, ^21^, ^22^, ^23^ ^]^ Unlike conventional metal batteries, where a thick metal anode is used, anode‐free batteries eliminate the need for a thick metal anode. Instead, during the charging process, metal ions from the electrolyte are plated directly onto a current collector and subsequently removed during the discharge process.^[^ ^20^, ^21^, ^22^, ^23^ ^]^ This approach provides several advantages, such as increasing the energy density by reducing the amount of inactive material, cost reduction, and improved safety by minimizing side reactions associated with excess metal.^[^ ^24^, ^25^ ^]^

**Scheme 1:**
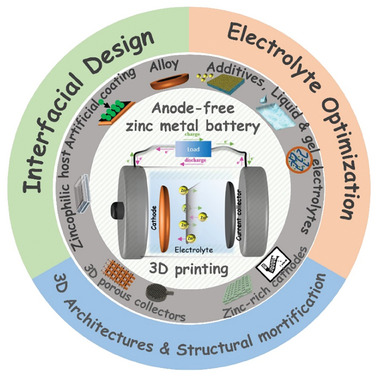
An overview of the proposed review, mainly focusing on interfacial design, electrolyte optimization, and architecture modifications via 3D printing to address the challenges for AFZMBs.

For instance, the concept of anode‐free batteries was first proposed by Neudecker et al. in the year 2000 for lithium metal batteries.^[^ ^20^ ^]^ To simplify the cell configuration and enhance the energy density, they demonstrated a novel thin film battery design consisting of a copper (Cu) anode current collector, a solid lithium electrolyte, and LiCoO_2_ as the cathode, thus avoiding the need for a pre‐existing lithium metal as anode. Instead, the battery operates with a lithium anode that is plated on a Cu anode current collector from the LiCoO_2_ cathode during the initial charging process. Subsequently, Li^+^ ions are stripped from the Cu anode during discharging and re‐inserted into the cathode and vice‐versa. As a result, this new concept, which eliminates the pre‐existing lithium metal anode, opens up new opportunities to enhance the energy density and address safety concerns. Nevertheless, the limited lithium reserve and high manufacturing costs continue to hinder the sustainable development of anode‐free lithium batteries. Very recently, various groups have explored the potential of extending this design to anode‐free sodium batteries, AFZMBs, anode‐free aluminum batteries, etc.^[^ ^24^, ^25^, ^26^, ^27^, ^28^, ^29^ ^]^ In this review, we discuss and analyze the recent progress in AFZMBs, focussing on key developments and the challenges that remain in this nascent area of research for practical implementation. Initially, we outline the conventional ZMBs and their challenges. Following in the subsequent sections, we examine the fundamental aspects of AFZMBs, including their working mechanism and advantages over conventional ZMBs. Furthermore, we explore recent strategies for attaining uniform Zn deposition, emphasizing modifying the current collector, which plays a crucial role in facilitating Zn plating. The electrolyte modification is also critically examined, which is essential for optimizing the AFZMBs. Finally, we present 3D printing as a promising strategy for fabricating advanced current collectors with improved structural properties and developing zinc‐rich cathodes, along with an outlook on the future perspective of AFZMBs (**Scheme** **1**).

## 2 Conventional Aqueous Zinc‐Metal Batteries (ZMBs) and Their Key Issues

In general, conventional aqueous‐based ZMBs are a class of rechargeable batteries that use Zn metal directly as the anode material. This is because Zn anode possesses certain merits, such as high safety over lithium, high theoretical gravimetric capacity (820 mAh g^−1^), and volumetric capacity (5855 mAh cm^−3^). Additionally, the redox potential of Zn metal is lower (−0.76 V vs SHE), highlighting its potential for high‐energy storage applications (Figure 1).^[^ ^30^, ^31^, ^32^ ^]^ However, it is still challenging to practically implement ZMBs with superior electrochemical performance, particularly in terms of stable cycling stability. This challenge arises mainly due to inherent issues related to Zn metal anode and parasitic reactions: i) uncontrollable dendritic formation; ii) hydrogen evolution reaction (HER) and continuous corrosion of Zn anode; iii) surface passivation, and low coulombic efficiency.^[^ ^30^, ^33^, ^34^ ^]^

### 2.1 Uncontrollable Dendritic Formation

**Figure 2:**
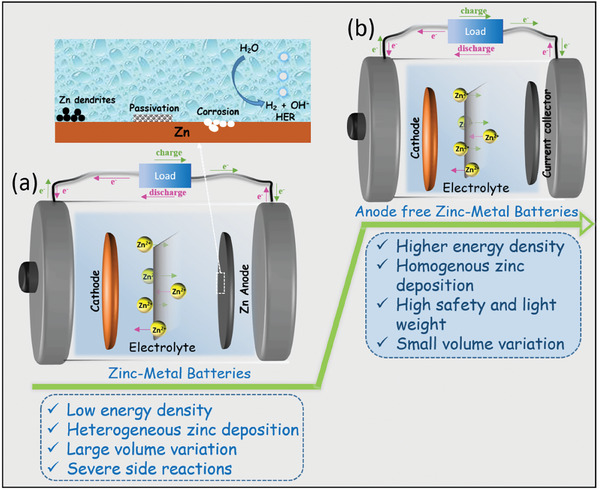
Schematics illustrations and overview of a) ZMBs, and b) AFZMBs.

In aqueous ZMBs, the formation of Zn dendrites on the anode surface poses a significant challenge for practical applications, primarily due to uneven Zn deposition.^[^ ^34^ ^]^ During initial charging, Zn^2+^ ions undergo reduction and are deposited on the Zn anode surface, whereas during discharging, Zn^2+^ ions are de‐intercalated from the anode, transformed into soluble Zn^2+^ ions, and migrate through the electrolyte toward the cathode due to the presence of electric field (**Figure** **2**a). However, the deposition and stripping of Zn^2+^ ions are not evenly distributed across the surface of the Zn anode during charging.^[^ ^34^ ^]^ Instead, Zn^2+^ ions tend to selectively accumulate at threading dislocations on the surface of the anode due to their influence on the distribution of the electric field. This uneven deposition results in the formation of Zn dendrites (Figure 2a), and as such, it can lead to short circuits or even battery failure.^[^ ^16^ ^]^ Therefore, advanced strategies, such as using 3D porous current collectors to promote uniform Zn deposition, have shown promise in suppressing dendrite growth.^[^ ^35^ ^]^

### 2.2 Hydrogen Evolution Reaction and Continuous Corrosion of Zn Anode

In addition to dendrite formation, severe unwanted side reactions also occur including HER, and corrosion of Zn metal anode during cycling.^[^ ^17^, ^18^, ^19^ ^]^ The HER, resulting from the reduction of water in an aqueous electrolyte, directly competes with the deposition of Zn^2+^ ions during charging and generates hydrogen gas as a by‐product. As a result, this side reaction reduces the active utilization of Zn for reversible reaction and lowers the coulombic efficiency while also exacerbating electrolyte depletion.^[^ ^17^, ^36^ ^]^ Additionally, the continuous production of hydrogen gas may result in internal pressure within the battery, potentially leading to leakage or swelling over time. Besides, corrosion further exacerbates this issue by generating by‐products such as ZnO or Zn(OH)_2_, which passivate the Zn surface, reducing its reactivity and blocking Zn deposition/stripping processes. Electrolyte engineering, including using super‐concentrated electrolytes or additives like ionic liquids, has been shown to suppress HER by reducing water activity.^[^ ^31^, ^36^ ^]^ Advanced separators and optimized electrolyte compositions can also mitigate these side reactions.

### 2.3 Surface Passivation and Low Coulombic Efficiency

Another major challenge in ZMBs is surface passivation and low coulombic efficiency, where an insulting layer forms on the surface of Zn anode during cycling, that typically includes by‐products like ZnO, Zn(OH)_2_ or ZnSO_4_[Zn(OH)_2_]_3_.xH_2_O depending on the electrolyte's pH.^[^ ^36^ ^]^ For instance, ZnO and Zn(OH)_2_ layers precipitate due to zincate ion supersaturation in alkaline aqueous electrolyte and ZnSO_4_[Zn(OH)_2_]_3_.xH_2_O form as a by‐product in acidic electrolyte. This layer hinders the flow of Zn^2^⁺ ions, increases interfacial resistance, and decreases the reversibility of Zn plating and stripping, thereby decreasing the battery's performance and reducing the coulombic efficiency and cycling stability.^[^ ^33^, ^34^, ^35^, ^36^ ^]^ Therefore, strategies such as SEI engineering and artificial protective layers have demonstrated the potential to mitigate passivation and improve the performance of ZMBs.^[^ ^31^, ^33^ ^]^

All these challenges collectively hinder Zn utilization, degrade electrochemical performance, and limit the cycle life of ZMBs. Therefore, in order to address these issues, there have been considerable attempts to mitigate the above challenges associated with ZMBs. This includes electrolyte engineering by optimizing the electrolyte composition and adding additives or introducing super concentrated electrolytes, choice of current collectors such as 3D porous host to suppress dendrite formation, artificial solid electrolyte interface (SEI) engineering to promote uniform Zn deposition, and as well as effectively suppresses Zn dendrite growth, choice of advanced separator, etc.^[^ ^30^, ^37^, ^38^, ^39^, ^40^, ^41^, ^42^, ^43^, ^44^ ^]^ While these practical strategies have significantly improved the development of ZMBs, a few bottlenecks still persist in fully unlocking the potential of ZMBs for practical applications such as the scalability of 3D anode hosts, the long‐term stability of artificial SEIs, and the compatibility of additives with various electrolytes remain unresolved. Furthermore, the excess Zn anode required in ZMBs impedes the energy density, promoting researchers to investigate novel alternatives and strategies to overcome these challenges.

## 3 Anode Free Zinc‐Metal Batteries, Working Principle and its Advantages Over Conventional ZMBs

**Figure 3:**
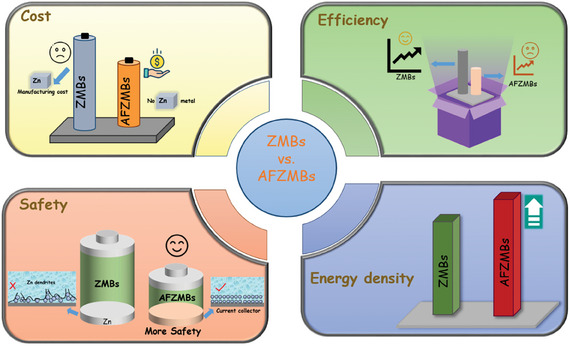
Comparison of ZMBs versus AFZMBs in terms of cost, safety, coulombic efficiency, and energy density.

AFZMBs based on aqueous electrolytes are considered a promising candidate for sustainable energy storage due to their high energy densities and reduced manufacturing costs by eliminating the pre‐existing Zn anode during the battery assembly. These designs provide a new pathway toward robust and sustainable energy storage, making it an emerging area of research to address the constant challenges encountered by conventional ZMBs.^[^ ^27^ ^]^ In contrast to conventional ZMBs that utilize excess Zn metal as the anode, AFZMBs depend on Zn^2+^ ions from the electrolyte to generate the active anode material during the charging process. This reduces the total Zn requirement, thereby optimizing the cell design while improving energy density by eliminating the necessity for a pre‐deposited Zn layer for energy storage applications where weight and space are critical.^[^ ^27^ ^]^Figure 2 shows the differences between conventional ZMBs and the AFZMBs. The AFZMBs consist of an anode current collector, electrolyte, and cathode material, while conventional ZMBs employ Zn metal as an anode, where Zn^2+^ ions are alternately stripped and deposited during charging and discharge cycles.^[^ ^30^, ^31^, ^32^ ^]^ In contrast, AFZMBs do not need Zn metal as an anode; instead, during charging, Zn^2+^ ions from the electrolyte are deposited electrochemically onto the current collector, i.e., at the anode (Figure 2b). During discharging, Zn^2+^ ions are extracted from the anode and intercalated into the cathode. Based on this configuration, the AFZMBs can offer several advantages over the conventional ZMBs, as shown in **Figure** **3**:

1. First, the absence of an anode in AFZMBs will reduce the battery's overall weight and volume, providing additional space for active materials and increasing its energy density. In contrast, the ZMBs can also be used for energy storage, but their heavy weight poses a problem in applications that require high energy density.
2. Second, the absence of an anode in AFZMBs reduces manufacturing costs. This simplification decreases the number of stages in the production process and removes the complexities during the battery assembly. As a result, the market penetration of AFZMBs is likely to be easier.
3. Thirdly, compared to ZMBs, AFZMBs may provide higher safety owing to the lack of a Zn metal anode compared to ZMBs. AFZMBs utilize a current collector, wherein Zn is electrochemically deposited during charging. However, such a design may still result in dendrite formation and side reactions. Therefore, several mitigation strategies, including surface modification, electrolyte engineering, and 3D hosts, will be discussed in the next section to mitigate these challenges. Furthermore, by removing the need for an excessive Zn metal anode and employing various modification strategies, AFZMBs enhance energy density and cycling stability while reducing battery weight and enabling higher energy storage, making them safer and more sustainable than ZMBs for a wide range of applications.^[^ ^27^ ^]^

## 4 Recent Advances in Anode Free Zinc‐Metal Batteries

Ever since the concept of anode‐free batteries was proposed, there has been a steady growth in the number of publications over the past few years related to anode‐free batteries.^[^ ^21^, ^22^, ^23^, ^24^, ^25^, ^26^, ^27^ ^]^ Among these, the immense potential of AFZMBs has led to significant progress over conventional ZMBs in terms of high energy density, suppressing Zn dendrites formation, optimizing electrolyte composition, and improving efficiency and cycling stability.^[^ ^27^, ^45^, ^46^, ^47^, ^48^, ^49^, ^50^, ^51^, ^52^, ^53^, ^54^, ^55^, ^56^ ^]^ In this section, we will summarize the recent strategies designed to enhance the performance of AFZMBs, focusing on key areas such as current collector modification and electrolyte optimization.

### 4.1 Current Collector Modification

The current collector plays a crucial role in the smooth functioning of the AFZMBs, as it serves as the substrate for the anode during Zn deposition without a conventional Zn metal anode. Therefore, optimizing the design of the current collector is crucial to improve the battery's performance. Ideally, for AFZMBs, the current collector should exhibit outstanding electrochemical stability, low nucleation overpotential, and a minimal surface diffusion barrier to facilitate uniform Zn deposition and suppress dendritic growth. To overcome these challenges, some important state‐of‐the‐art strategies have been recently proposed to improve the reversibility of Zn plating/stripping for high‐energy AFZMBs, including surface modification, 3D structured frameworks, zincophilic engineering, and polymer‐based surface modifications.

#### 4.1.1 Surface Modification

**Figure 4:**
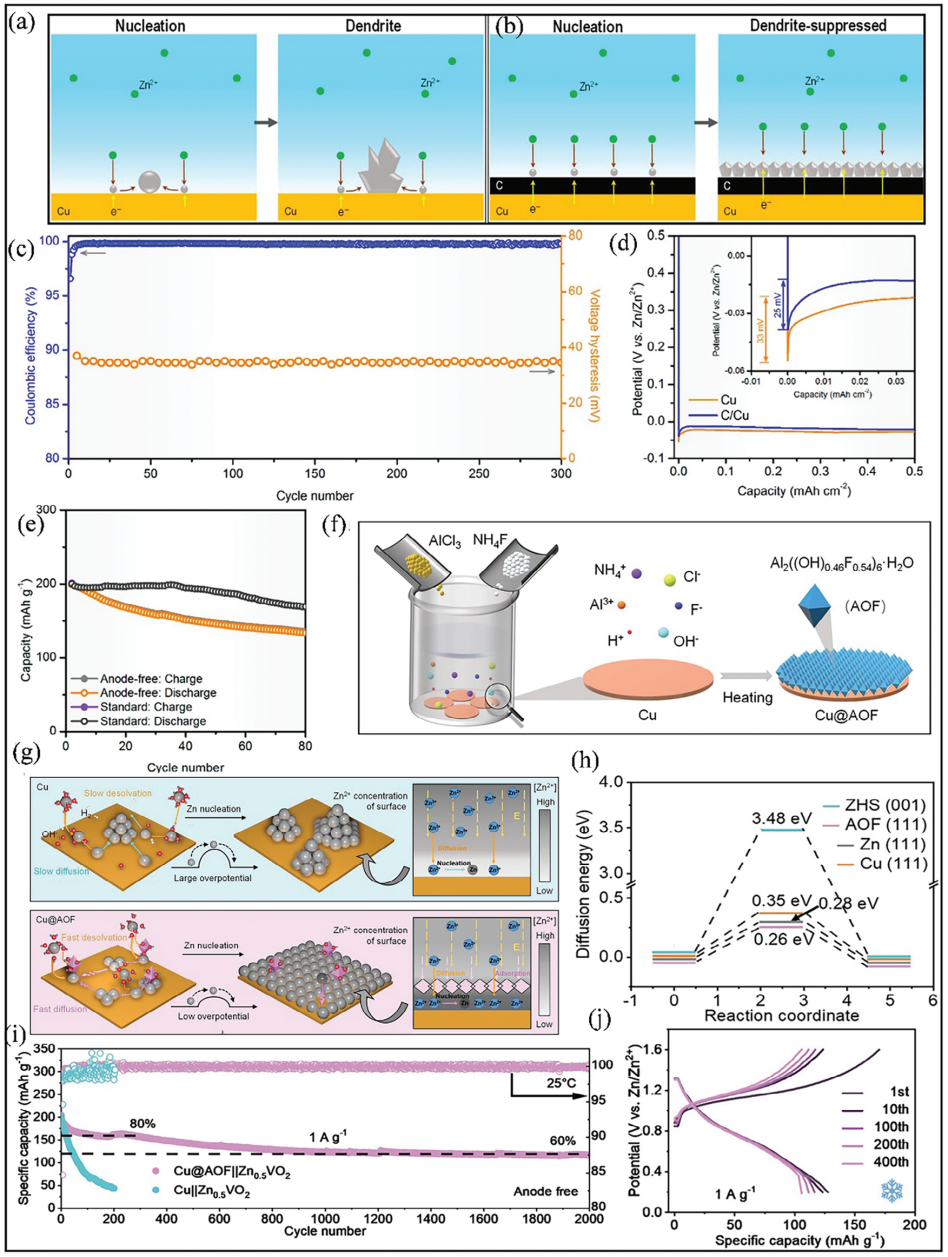
Strategies to mitigate dendrite formation via surface engineering. a) Schematics illustration of Zn nucleation behavior on (a) bare Cu and b) modified C/Cu current collector for AFZMBs. c) Cycling stability of C/Cu during the Zn plating/stripping process in 3 m Zn(CF_3_SO_3_)_2_ at a current density of 1 mA cm⁻^2^ and 0.5 mAh cm⁻^2^ plating capacity. d) Comparison of galvanostatic Zn plating profiles of Cu and modified Cu (C|Cu), with the inset highlighting the initial nucleation overpotential. e) cycling stability of anode‐free Zn‐MnO_2_ battery at a current density of 1 mA cm⁻^2^. Reproduced with permission.^[^ ^27^ ^]^ Copyright 2021, American Chemical Society. f) Schematic representation of the synthesis process of Cu@AOF. g) Schematic illustrations of Zn plating on bare Cu and Cu@AOF. h) Comparison of diffusion energy barriers for ZHS (001), AOF (111), Zn (111), and Cu (111). i) Long‐term cycling stability test at a current density of 1 A g^−1^. j) Galvanostatic charge/discharge profiles of the assembled cell Cu@AOF ||Zn_0.5_VO_2_ at −20 °C. Reproduced with permission.^[^ ^45^ ^]^ Copyright 2023 Wiley‐VCH GmbH.

Surface modification has been considered a promising strategy for AFZMBs to regulate uniform Zn deposition and mitigate unwanted side reactions at the electrode/electrolyte interface.^[^ ^27^, ^45^, ^46^ ^]^ For example, in the year 2021, Zhu et al. designed the first AFZMBs in aqueous electrolyte.^[^ ^27^ ^]^ This study demonstrates an anode‐free configuration, which eliminates using Zn metal as an anode by simply utilizing a nucleation layer of carbon nanodiscs onto the Cu current collector. These nucleation layers protect the anode surface from side reactions and provide accessible nucleation sites for Zn deposition. In addition, this protective coating layer can improve the stability of the Zn nucleation and mitigate dendritic growth on the surface. As shown in **Figure** **4**a, the pristine Cu current collector exhibits uneven Zn deposition whereas the modified current collector coated with carbon nanodiscs (abbreviated as C/Cu) displays a lower nucleation potential and exhibits a homogenous distribution of Zn^2+^ flux (Figure 4b).^[^ ^27^ ^]^ As a result, the Zn plating/stripping process on C/Cu exhibits a high initial coulombic efficiency of 99.6% over 300 cycles at a current density of 1 mA cm^−2^ in 3 M Zn(CF_3_SO_3_)_2_ aqueous electrolyte (Figure 4c). The comparison of Zn plating overpotential at the same current density is also shown inFigure 4d. The results demonstrate that the modified C/Cu current collector displays the lowest Zn plating overpotential of 25 mV in comparison to pristine Cu (33 mV). This substantial decrease in overpotential is likely attributed to the low lattice mismatch between the two interfaces.^[^ ^47^, ^48^, ^49^, ^50^, ^51^ ^]^ Furthermore, an anode‐free cell is also assembled using pre‐zincification MnO_2_ as a cathode in 3 M Zn(CF_3_SO_3_)_2_ aqueous electrolyte. The assembled anode‐free based Zn‐MnO_2_ battery delivered an energy density of 135 Wh kg^−1^ with a capacity retention of 68.2% over 80 cycles (Figure 4e) compared to standard ZMBs. While this study suggests the potential for an anode‐free Zn configuration, long‐term cycling stability with high coulombic efficiency remains a formidable task. Recently, MXene‐based current collectors have shown considerable advantages in improving the reversibility of Zn deposition by facilitating the formation of a ZnF_2_‐SEI in AFZMBs, which may be a promising approach for achieving high energy storage systems.^[^ ^52^, ^53^ ^]^

Another alternative strategy for achieving uniform Zn deposition and inhibiting dendritic growth in AFZMBs is by introducing functional coating materials on the Cu current collector. For instance, Wang et al. demonstrated that an aluminum hydroxide fluoride (AOF) layer coated on Cu current collector (Cu@AOF) exhibits zincophilic sites that facilitate uniform Zn deposition and mitigate most of the side reactions, thereby improving the electrochemical performance of AFZMB.^[^ ^45^ ^]^Figure 4f illustrates the synthesis procedure for preparing AOF on a Cu current collector, abbreviated as Cu@AOF. The authors found that AOF not only promotes uniform Zn deposition but also boosts the Zn^2+^ desolvation due to its strong affinity to water as well as reduces the diffusion energy barriers for Zn adatoms to facilitate rapid Zn diffusion and Zn growth as illustrated inFigure 4g and supported by density functional theory (DFT) calculations (Figure 4h). Subsequently, an AFZMB with Zn_0.5_VO_2_ was assembled, demonstrating a discharge capacity of 117 mAh g^−1^ over 2000 cycles at a current density of 1 A g^−1^ with a high coulombic efficiency of almost 100% (Figure 4i).^[^ ^45^ ^]^ Besides, the Cu@AOF|| Zn_0.5_VO_2_ cell also exhibited a low voltage polarization and excellent cycling stability at a very low temperature of –20 °C with a coulombic efficiency of 99.94% at a current density of 1 A g^−1^ (Figure 4j). This work suggests that AOF coatings could improve the Zn deposition reversibility and ensure long‐term stability under extreme cold conditions, making it a promising strategy for next‐generation AFZMBs.

#### 4.1.2 3D‐Structured Frameworks

**Figure 5:**
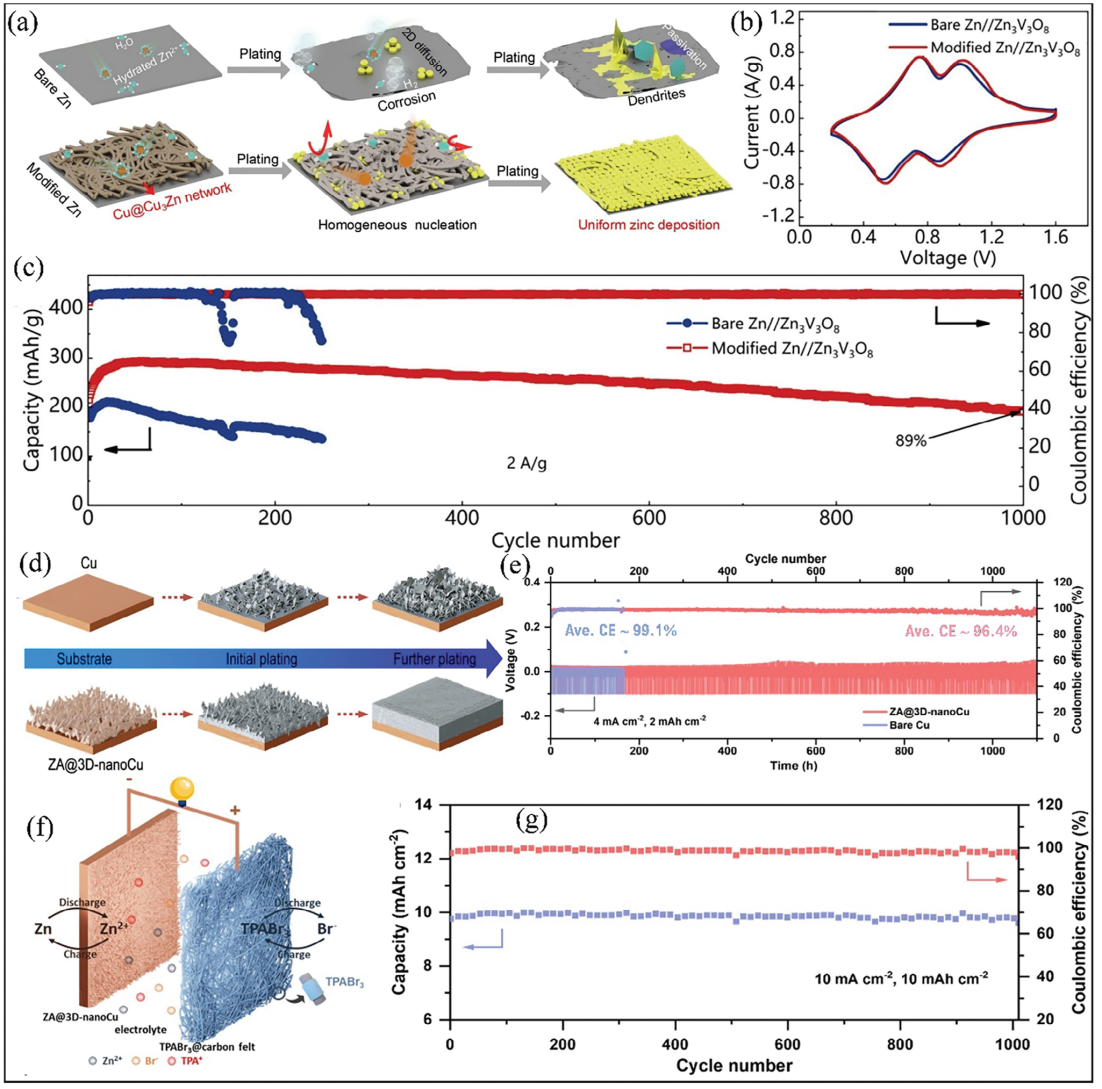
Schematic illustration of the 3D structured current collector introduced by zincophilic material. a) Schematic illustrations of Zn plating behavior on the surface of bare Zn and modified Zn anode. b) Cyclic voltammetry curves of the bare Zn versus modified Zn anode with Zn_3_ V_3_O_8_ as the cathode at a scan rate of 0.5 mV s^−1^. c) long‐term cycling stability in 3 m Zn(CF_3_SO_3_)_2_ aqueous electrolyte at a current density of 2 A g^−1^. Reproduced with permission.^[^ ^55^ ^]^ Copyright 2022, Elsevier. d) Schematic illustrations of Zn plating on bare Cu and modified ZA@3D‐nanoCu anode. e) Galvonstatic cycling profiles of ZA@3D‐nanoCu|Zn and Cu|Zn half cells in 2 m ZnBr_2_. f) Schematic diagram of the assembled ZA@3D‐nanoCu|Br_2_ battery, and g) Cycling stability of the assembled ZA@3D‐nanoCu|Br_2_ battery. Reproduced with permission.^[^ ^56^ ^]^ Copyright 2023, Elsevier.

3D‐based current collectors with a large surface are also an effective strategy to achieve dendrite‐free anodes with uniform Zn deposition.^[^ ^35^ ^]^ Unlike 2D planar current collectors, 3D‐structured offers abundant nucleation sites, reduces the local current, and mitigates volume variation during the cycling process, thereby improving the electrochemical performance of AFZMBs.^[^ ^35^ ^]^ Recently, it was reported that a 3D‐based current collector with zincophilic materials could significantly improve stability with high coulombic efficiency by lowering the nucleation barrier and inhibiting dendrite formation.^[^ ^54^, ^55^ ^]^ A 3D conductive Cu@Cu_3_ Zn alloy network for AFZMBs was designed by Dong et al. by subjecting Cu nanowires to a gas‐solid reaction at high temperature with Zn vapor and then modulating with a 3D network using a vacuum filtration technique.^[^ ^55^ ^]^ The authors found that the 3D conductive Cu@Cu_3_ Zn alloy as zincophilic sites not only decreases the plating overpotential but also promotes uniform Zn deposition and eliminates side reactions (**Figure** **5**a) compared to conventional Zn anode. As shown in Figure 5b, the AFZMBs assembled with Zn_3_ V_3_O_8_ as cathode shows similar pairs of redox peaks in 3 M Zn(CF_3_SO_3_)_2_ aqueous electrolyte. In addition, the assembled cell with 3D conductive Cu@Cu_3_ Zn alloy as anode exhibits a capacity retention of 89% after 1000 cycles at a current density of 2 A g^−1^ with a coulombic efficiency of 100%. In contrast, the conventional ZMBs assembled with bare Zn//Zn_3_ V_3_O_8_ show inferior cycling stability (Figure 5c).^[^ ^55^ ^]^ Similarly, Chen and co‐workers designed a 3D zincophilic nano‐copper (ZA@3D‐nanoCu) with strong binding energy, which facilitates homogenous deposition and stripping of Zn metal, as shown inFigure 5d. It is worth mentioning here that incorporating zincophilic materials into the current collector is a promising strategy for promoting uniform Zn^2+^ ion distribution, reducing the nucleation barrier, and improving the stability and performance of AFZMBs.^[^ ^54^, ^55^, ^56^ ^]^ To examine the superiority of ZA@3D‐nanoCu over conventional Zn anode, the authors test the electrochemical activity of the ZA@3D‐nanoCu|Zn and Cu|Zn half cells. As shown inFigure 5e, the Zn deposition and stripping coulombic efficiency of ZA@3D‐nanoCu|Zn can reach 98.4% over 1100 h at an areal capacity of 2 mAh cm^−2^ and current density of 4 mA cm^−2^. In contrast, the Cu|Zn can exhibit a lifespan of only 160 h. Subsequently, an anode‐free Zn‐Br_2_ cell is assembled to check the feasibility of ZA@3D‐nanoCu for practical application with Br_2_ as the cathode.^[^ ^56^ ^]^ As shown in Figure 5f, Zn is electrochemically deposited onto the ZA@3D‐nanoCu electrode during the charging process, while bromide ions (Br⁻) undergo a chemical reaction to become bromine (Br_2_). Subsequently, bromine reacts with TPABr on the cathode to produce TPABr_3_. During the discharge process, these reactions undergo a reversal. Based on this, the anode‐free Zn‐Br_2_ battery exhibits outstanding cycling stability with a capacity retention of 99%, maintaining a capacity of 10 mAh cm⁻^2^ over 1000 cycles, with a coulombic efficiency of 98.95% (Figure 5g). Recently, Huang et al. proposed a novel 3D zincophilic substrate with silver aerogel (AgNWA) as an anode for AFZMBs.^[^ ^57^ ^]^ Benefitting from the high binding energy of silver, the authors demonstrated an ultrahigh‐rate Zn deposition at 40 mA cm^−2^ with an areal capacity of 10 mAh cm^−2^ over 200 cycles and achieved a coulombic efficiency of 99.8%. The 3D AgNWA not only provides a high surface area but also makes it easier for Zn deposition by uniformly distributing the electric field without any volume variation during the cycling process compared to a 2D planar current collector.^[^ ^57^ ^]^ To evaluate the feasibility of 3D AGNWA, an AFZMBs was assembled with pre‐zincificated MnO_2_ cathode in an aqueous electrolyte. The assembled AFZMB displays a specific capacity as high as 230 mAh g^−1^ at a current density of 0.5 A g^−1^ with a capacity retention of 73% after 600 cycles.^[^ ^57^ ^]^ It is to be noted that, unlike the 2D planar current collector, where significant Zn loss could be observed during the cycling process. This work hints that one can achieve uniform Zn deposition and stripping with minimal Zn loss during cycling, making it a promising strategy for designing advanced electrodes for high‐energy storage applications.

#### 4.1.3 Zincophilic Engineering

Apart from the aforementioned strategies, surface modification with zincophilic materials has received much attention in AFZMBs to improve the Zn deposition and mitigate the side reactions. This may be attributed to the reduced nucleation overpotential induced by introducing zincophilic materials (such as Ag, Sn, Sb, Au, etc.) on the current collector, resulting in homogeneous Zn deposition and stripping. It also enhances Zn affinity and modulates Zn^2^⁺ flux, resulting in improved cycling stability and coulombic efficiency.^[^ ^54^, ^58^, ^59^ ^]^

**Figure 6:**
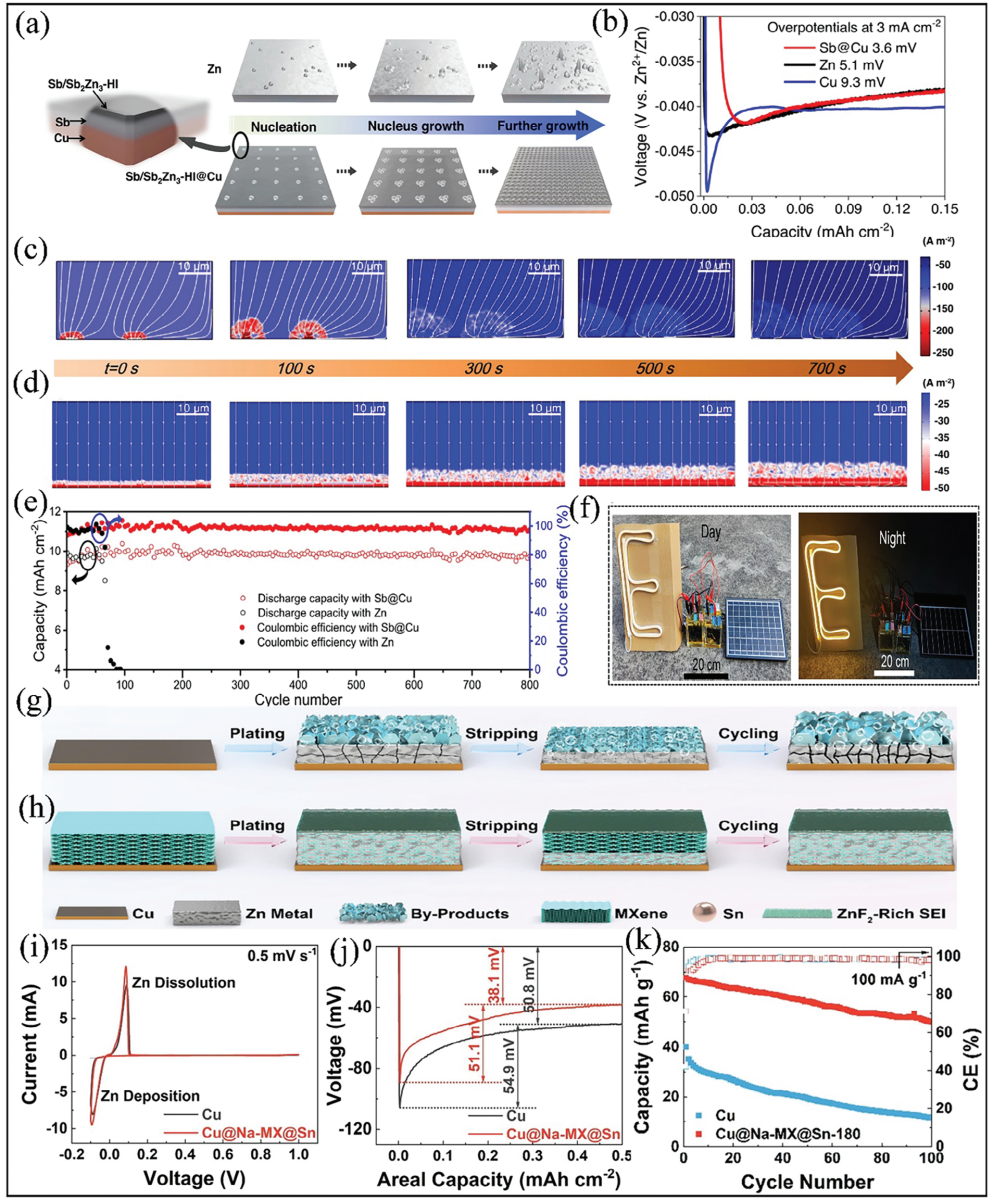
Strategies for the zincophilic materials by integrating 2D materials. a) Zn plating and nucleation behavior on two different surfaces, i.e., bare Zn and modified zincophilic Sb/Sb_2_Zn_3_@HI. b) Nucleation overpotential profiles on Zn, Cu, and Sb@Cu substrates, during Zn electrodeposition at a current density of 3 mA cm^−2^ in 2 M ZnBr_2_ electrolyte. Illustrations of stimulated analysis of current density distribution during Zn plating on c) bare Zn, d) Sb/Sb_2_Zn_3_@HI substrates at room temperature. e) Cycling stability of anode‐free Zn‐Br_2_ battery with bare Zn and Sb/Sb_2_Zn_3_@HI at a current density of 10 mAh cm^−2^ and 10 mA cm^−2^, and f) digital images of the assembled Zn‐Br_2_ battery with solar powered panel lighting at day and night. Reproduced with permission.^[^ ^60^ ^]^ Copyright 2023, Springer Nature. Zn plating behavior on g) bare Cu and h) modified Cu@Na‐MX@Sn substrates, respectively. i) Cyclic Voltammetry profiles of the assembled asymmetric cells with bare Cu and modified Cu@Na‐MX@Sn substrates at a scan rate of 0.5 mV s^−1^. j) Nucleation overpotential profiles on bare Cu and modified Cu@Na‐MX@Sn at a current density of 1 mA cm^−2^. k) Cyclic stability at a current density of 10 mA g^−1^. Reproduced with permission.^[^ ^61^ ^]^ Copyright 2023, Elsevier.

Therefore, incorporating zincophilic materials would be a promising strategy to achieve homogenous Zn deposition and stripping of Zn metal. For example, Zheng et al. investigated the Zn deposition and stripping process by designing a robust 2D heterostructured interface (Sb/Sb_2_Zn_3_@HI), with the goal of improving the areal capacity and coulombic efficiency for AFZMBs.^[^ ^60^ ^]^ **Figure** **6**a shows the schematic illustration of Zn deposition on two different substrates. As shown, the 2D interface with zincophilic layer (Sb/Sb_2_Zn_3_@HI) on Cu substrate promotes more uniform Zn nucleation and has a high nucleation rate for Zn deposition and stripping. In contrast, the Zn foils without a zincophilic layer induce inhomogeneous Zn deposition, which can lead to dendrite formation and short circuits. Moreover, Sb/Sb_2_Zn_3_@HI exhibits lower nucleation overpotential than that of alternative current collectors, i.e., Zn or Cu substrate. As demonstrated in Figure 6b, the Sb/Sb_2_Zn_3_@HI shows a lower nucleation overpotential of 3.6 mV, whereas 5.1 and 9.3 mV overpotential were observed in the case of Zn and Cu substrates. This is further supported by calculating the adsorption energy barriers of Zn atoms on Zn (100) and Sb (104) planes by DFT and COMSOL simulations. It was found that the adsorption energy of Zn atoms on Sb (104) is lower (−3.12 eV) than the adsorption energy of Zn (100) planes, i.e., ‐0.72 eV. This indicates that zincophilic surface modification on the current collector can be a promising strategy for designing advanced electrodes for AFZMBs. Furthermore, COMSOL simulations were performed to understand the homogenous distribution of Zn and subsequent growth. As demonstrated inFigure 6c, inhomogeneous distribution of Zn and higher nucleation barriers could be observed on Zn substrate during the Zn plating process, thereby leading to a root‐tip predominance of Zn dendrite growth while Sb/Sb_2_Zn_3_@HI substrate exhibits homogenous current density and Zn^2+^ ion flux during the Zn plating process (Figure 6d) and promotes dendrite‐free deposition of Zn.^[^ ^60^ ^]^ The authors also assembled an anode‐free Zn‐Br_2_ cell to demonstrate the feasibility of Sb/Sb_2_Zn_3_@HI for practical applications. The anode‐free Zn‐Br_2_ battery delivered a higher areal capacity of 10 mAh cm^−2^ over 800 cycles with almost no capacity decay at a current density of 10 mA cm^−2^_,_ whereas the battery assembled without zincophilic material shows inferior cycling stability and drops before 100 cycles (Figure 6e). This again suggests that constructing a robust 2D heterostructured interface on current collectors could be a promising strategy to achieve dendrite‐free AZMBs. Furthermore, the assembled Zn‐Br_2_ battery is combined with a renewable energy source, such as a solar panel (shown in Figure 6f), to light an LED display. The configuration is depicted both during the day and at night to emphasize its practicability. Although the above‐mentioned strategies have been developed for current collectors, the development of interfacial designs on the anode substrate remains essential to enhance the uniformity of Zn deposition.

Li et al. recently engineered a heterointerface by integrating 2D metal carbide nanosheets (Na‐MXene) with 0D metal nanodots (Sn) for AFZMBs.^[^ ^61^ ^]^ The Na‐MXene with 0D metal nanodots (Sn), i.e., Na‐MX@Sn, is synthesized by a series of electrostatic self‐assembly and then followed by a chemical reduction method. The authors demonstrated that the modified Na‐MX@Sn coated on Cu current collector significantly reduces the Zn nucleation barrier, promoting uniform Zn deposition and controlled crystal growth compared to conventional current collectors. As shown inFigure 6g, compared with conventional Cu foil, the modified Na‐MX@Sn coated on Cu current collector, i.e., Cu@Na‐MX@Sn, exhibits homogenous deposition and stripping of Zn metal (Figure 6h), which lowers the Zn^2+^ ion flux and reduces the nucleation overpotential during cycling with no sign of dendrite formation. This may be attributed to the introduction of Sn nanodots on Na‐MX and the stable ZnF_2_‐rich SEI layer formed on the surface.^[^ ^59^, ^61^ ^]^ Figure 6i shows the CV profiles for asymmetrical cells with Zn||Cu and Zn||Cu@Na‐MX@Sn. It can be noticed that the onset potential voltage is slightly lower for Zn||Cu@Na‐MX@Sn than that of Zn||Cu cell, suggesting a more homogenous distribution of the Zn plating/stripping process. In addition, the Cu@Na‐MX@Sn can facilitate Zn^2+^ ion migration and minimize volume changes during cycling, while the uniformly distributed Sn nanodots can regulate the Zn nucleation behavior.^[^ ^61^ ^]^ As a result, the Cu@Na‐MX@Sn exhibits a reduced nucleation and mass transfer overpotential of 51.1 and 38.1 mV than that of conventional Cu substrate, i.e., 54.9 and 50.8 mV (Figure 6j). Besides, AFZMBs were assembled in pairs with LiMn_2_O_4_ as a cathode, demonstrating a higher discharge capacity of 40.18 mAh g^−1^ at 100 mA g^−1^ even after 100 cycles with a capacity retention of 68.9%.^[^ ^61^ ^]^ This improved electrochemical performance may be attributed to the uniform electric field generated by MXene, the zincophilic properties of Sn, which guide the Zn nucleation, and the stable ZnF_2_ ‐richSEI formed on the surface.^[^ ^61^ ^]^ In contrast, the conventional Cu foil assembled with LiMn_2_O_4_ shows inferior cycling stability with a discharge capacity of only 11.65 mAh g^−1^ after 100 cycles (Figure 6k).^[^ ^61^ ^]^

#### 4.1.4 Polymer‐Based Surface Modifications

**Figure 7:**
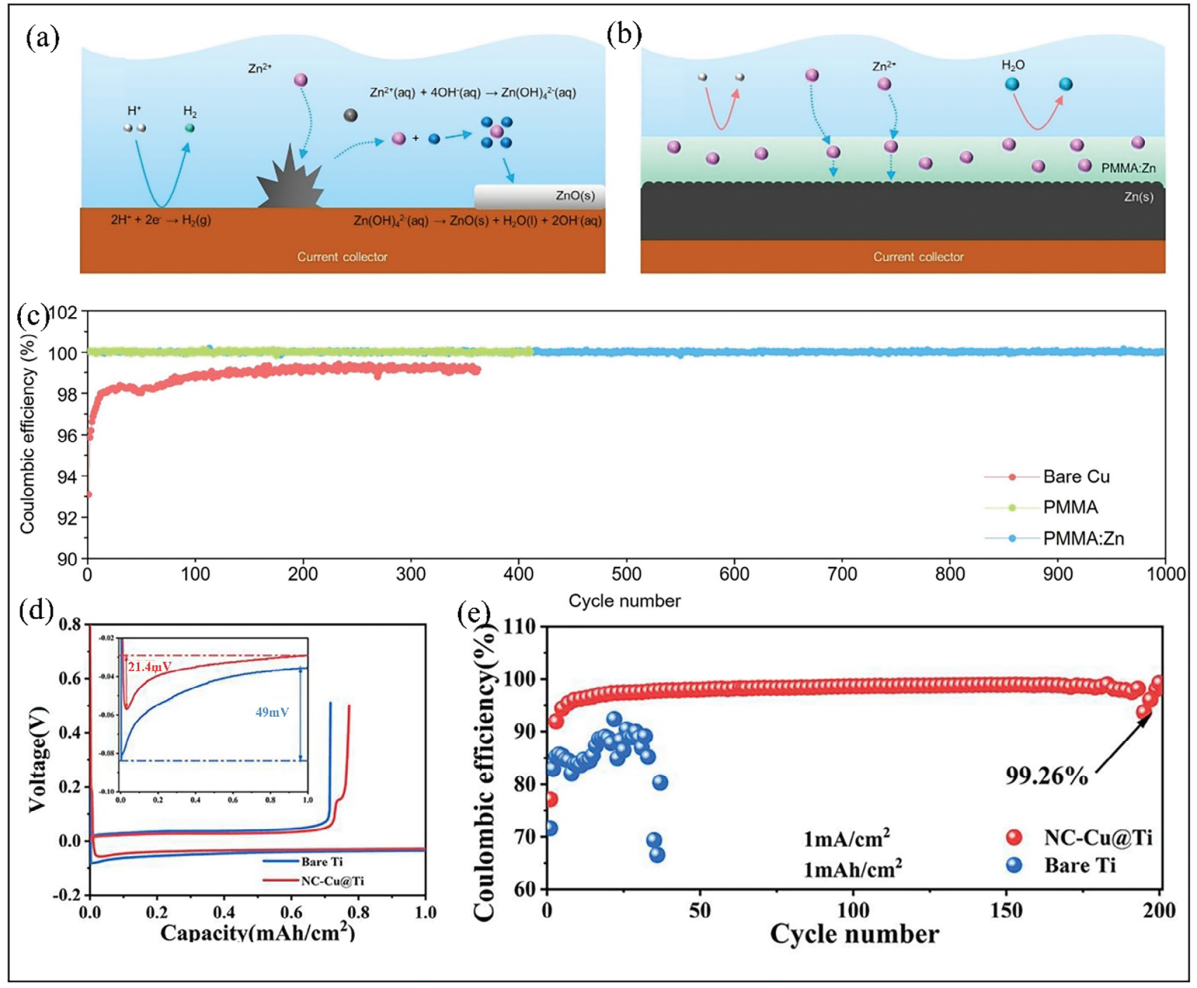
Strategies to control interfacial reactions during Zn plating. a) Schematic representation of typical challenges faced during Zn plating on bare Cu. b) Zn plating and protection mechanism of PMMA: Zn coating layer. c) Cycling stability and coulombic efficiency of the half cells during Zn plating/stripping at a current density of 1 mA cm^−2^. Reproduced with permission.^[^ ^63^ ^]^ Copyright 2023 Elsevier. d) Initial Zn plating/stripping profiles of bare Ti and modified NC‐Cu@Ti‐0.5 substrate. Inset Figure 3d shows the nucleation potential on bare Ti and modified NC‐Cu@Ti‐0.5, and e) Cycling stability and coulombic efficiency of bare Ti and modified NC‐Cu@Ti‐0.5. Reproduced with permission.^[^ ^64^ ^]^ Copyright 2024 American Chemical Society.

Recently, polymer‐based materials have become an essential strategy for modifying current collectors for ZMBs.^[^ ^62^, ^63^ ^]^ This method enhances the electrochemical stability and ensures uniform Zn deposition on the Zn anode surface by suppressing the growth of Zn dendrites. For instance, Park and co‐workers proposed that implementing a protective layer on the Cu surface can significantly improve the electrochemical performance, suppress the formation of Zn dendrites, and reduce side reactions.^[^ ^63^ ^]^ Compared with the bare Cu current collector (unprotected layer), as shown in **Figure** **7**a, this protective layer PMMA:Zn consists of a dense polymethylmethacrylate (PMMA) matrix infused with zinc ions, which is spin‐coated on the surface of the Cu current collector (Figure 7b).^[^ ^63^ ^]^ The PMMA: Zn (protected layer) can mitigate the side reactions or formation of by‐products by preventing direct contact between the electrode and electrolyte interface and as a result of the PMMA: Zn/Cu half cells achieved high coulombic efficiency of almost 100% with outstanding cycling stability at a current density of 10 mA cm^−2^ (Figure 7c). AFZMBs assembled with PMMA: Zn/Cu as anode and ZnMnO_2_ cathode exhibited a discharge capacity of 257 mAh g^−1^ at a current density of 1 A g^−1^ and retained more than 80% of its capacity after 300 cycles. In the case of bare Cu without polymer surface modification, it shows inferior cycling stability and completely fails after 50 cycles, while the PMMA/Cu cell shows an initial discharge capacity of 230 mAh g^−1^; however, it sharply decreased to 11 mAh g^−1^ after 100 cycles, indicating that PMMA was not entirely suitable to prevent the formation of dendrites.^[^ ^63^ ^]^ To improve the stability of AFZMBs with higher coulombic efficiency. Jing et al. adopted a simple co‐electrodeposition technique to produce nanocrystalline Cu onto titanium foil (NC‐Cu@Ti).^[^ ^64^ ^]^ This design is intended to improve the stability and performance of AZMBs. The authors demonstrated that by varying the concentration of Cu^2+^ ion during the electrodeposition, the structure and morphology of Cu crystals formed on NC‐Cu@Ti may play a vital role in regulating the nucleation sites for Zn deposition and mitigating side reactions for AFZMBs.^[^ ^64^ ^]^ Through experimental studies, it was found that the NC‐Cu@Ti‐0.5 facilitates rapid electron transport and higher binding sites for Zn^2+^ ions at the interface by reducing undesired interfacial side reactions and corrosion.^[^ ^64^ ^]^ As shown in Figure 7d, the NC‐Cu@Ti‐0.5 exhibited a lower overpotential of 21.4 mV compared to the bare Ti electrode of 49 mV. This indicates that NC‐Cu@Ti‐0.5 electrode can contribute to a more stable state for Zn deposition. In addition, the NC‐Cu@Ti‐0.5 demonstrated a high coulombic efficiency of 99.26% over 200 cycles. In contrast, the bare Ti sustained only 30 cycles (Figure 7e). Moreover, to check the feasibility of NC‐Cu@Ti‐0.5 for practical applications, the authors constructed AZMBs with MnO_2_ as a cathode, which could retain 80% of its discharge capacity. Furthermore, the assembled NC‐Cu@Ti‐0.5//MnO_2_ offers superior electrochemical performance and better rate capability in comparison to standard Zn//MnO_2_.^[^ ^64^ ^]^

As a result, this work hints that interfacial chemistry on the current collector can be a promising strategy for AFZMBs as it can eliminate side reactions, improve Zn^2+^ ion binding sites, and facilitate more uniform and stable Zn deposition. Furthermore, it is crucial to focus on reducing the weight of the current collectors in order to improve the energy density. Therefore, further exploration of advanced current collectors specifically on the interfacial chemistry, zinchophilicity, pore structure, and how these characteristics impact the process of nucleation and subsequent plating/stripping behavior are highly desired. All these investigations will offer significant knowledge about the intricate mechanisms that control the performance of current collectors in AFZMBs. It is noted here that when selecting a current collector for AFZMBs, several key factors are crucial: a) it must have high electronic conductivity and chemical stability to enable fast electron transfer and prevent corrosion; b) it should withstand a wide temperature range, c) mechanically stable, promote strong adhesion with Zn deposits for uniform deposition, and d) lightweight and cost‐effective to maximize energy density.

### 4.2 Electrolyte Modification

One of the major challenges in AFZMBs is the instability of Zn plating/stripping, which results due to uncontrolled Zn nucleation, and parasitic side reactions. Although traditional aqueous electrolytes offer high ionic conductivity, they often suffer from excessive free water molecules that react with Zn, leading to low coulombic efficiency and rapid capacity fading.^[^ ^65^ ^]^ To address these issues, identifying an appropriate electrolyte for the advancements of AFZMBs is highly desired. Studies suggest incorporating additives and introducing novel electrolytes can significantly improve the aforementioned challenges. The following subsections explore these key areas:

#### 4.2.1 Interfacial Engineering and Electrolyte Additives

**Figure 8:**
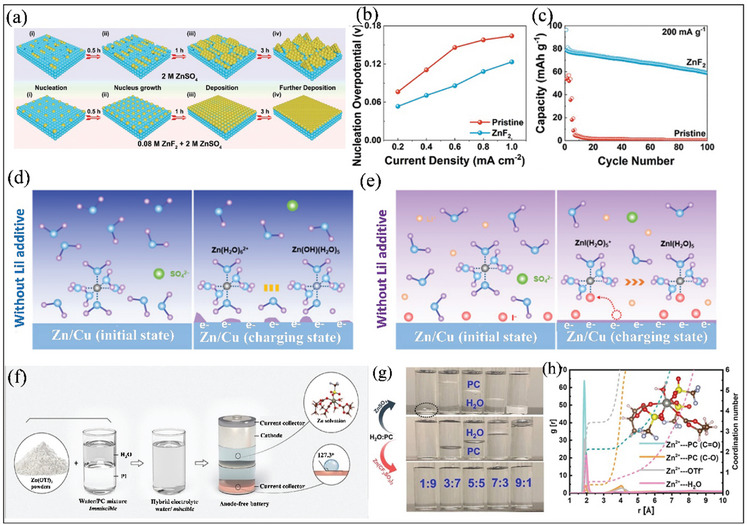
Strategies for the modification of electrolytes. a) Schematic representations for Zn plating at pristine electrolytes, i.e., 2 M ZnSO_4_ and additive ZnF_2_‐based electrolytes. b) Nucleation overpotential of Zn plating with different current densities. c) Cycling stability of the assembled cells using stainless steel and LiMn_2_O_4_ at a current density of 200 mA g^−1^ in additive ZnF_2_‐based electrolytes. Reproduced with permission.^[^ ^66^ ^]^ Copyright 2021 Wiley‐VCH GmbH. Schematic diagram illustrating the Zn^2^⁺ charge transfer behavior d) without LiI, and e) with modified LiI additive aqueous electrolyte. Reproduced with permission.^[^ ^68^ ^]^ Copyright 2024 The Royal Society of Chemistry. f) Schematic representation of the preparation of hybrid electrolyte, and highlighting its impact of hydrophobic protective interphase for Zn solvation structure. g) Digital images of water/PC mixture with different concentrations. h) Radial distribution function of Zn^2+^ solvation in hybrid Electrolyte. Reproduced with permission.^[^ ^71^ ^]^ Copyright 2022 American Chemical Society.

Introducing additives into the aqueous electrolytes is a vital strategy to improve the performance of AFZMBs, as these additives can significantly reduce the free water activity in aqueous electrolytes, thereby mitigating unwanted side reactions.^[^ ^65^ ^]^ For example, Feng et al. demonstrated that introducing interfacial engineering by adding a ZnF_2_ additive to an aqueous electrolyte (2 M ZnSO_4_) led to more uniform Zn deposition for AFZMBs in comparison to pristine electrolyte, as shown in **Figure** **8**a. This improvement is attributed to the addition of 0.08 M ZnF_2_ additive, which facilitates the formation of a stable F‐rich interfacial layer on the stainless steel current collector.^[^ ^66^ ^]^ In other words, this interfacial layer not only regulates the uniform Zn^2+^ distribution but also promotes uniform Zn deposition while suppressing side reactions such as HER and Zn corrosion.^[^ ^66^ ^]^ As a result, the ZnF_2_‐based electrolyte exhibited relatively low nucleation over various current densities compared to the pristine aqueous electrolyte (Figure 8b). Furthermore, the ZnF_2_‐based electrolyte shows a coulombic efficiency of 99.87% in AFZMBs.^[^ ^66^ ^]^ To check the feasibility of this electrolyte for practical applications, an anode‐free cell was assembled using LiMn_2_O_4_ as the cathode and stainless steel as the anode with 0.08 M ZnF_2_ + 2 M ZnSO_4_ aqueous electrolyte. As shown in Figure 8c, the assembled AFZMBs delivered a discharge capacity of 59 mAh g^−1^ over 100 cycles.^[^ ^66^ ^]^ Similarly, iodide ions (I^−^) have been identified as a promising electrolyte additive for regulating the initial stage of Zn nucleation and growth process in AFZMBs.^[^ ^67^, ^68^ ^]^ Shi et al. demonstrated that incorporating lithium iodide (LiI) as an additive into the aqueous electrolyte significantly enhances the Zn nucleation and deposition process.^[^ ^68^ ^]^ This is because I^−^ ions can facilitate the formation of a stable I^−^‐electrochemical double layer, which modulates two primary functions: First, it regulates the distribution of Zn^2+^ ions by reducing the energy barrier for nucleation, thereby promoting uniform zinc deposition and dissolution; and second, it minimizes the side reactions and surface passivation. Experimental and theoretical studies further revealed that in pristine ZnSO_4_ electrolyte, the initial Zn deposition occurs through a water‐rich Helmholtz layer on the Cu substrate (Figure 8d), resulting in slow reaction kinetics due to high Marcus charge transfer energy barriers and irregular deposition patterns. However, when LiI is added, the I^−^ ions form a I^−^‐rich Helmholtz layer that significantly reduces the energy barrier, enabling faster electron transfer through Zn‐I bonds (Figure 8e). This I^−^‐regulated process leads to preferred crystal growth orientation with dominant Zn (002) planes, ultimately achieving highly reversible Zn deposition/stripping suitable for high‐performance AFZMBs.^[^ ^68^ ^]^ Chen et al. introduced 1,3‐Dimethyl‐2‐imidazolidinone as an electrolyte additive that promotes the in‐situ formation of a bilayer SEI on the Zn surface, consisting of a mechanically robust layer on one side and the other chemically stable layer on the other side.^[^ ^69^ ^]^ This bilayer SEI is composed of ZnS and ZnCO_3_, respectively. By forming a strong bond with the Zn surface, the amorphous ZnS‐rich inner layer facilitates uniform Zn plating. It even protects the Zn surface from direct contact with the electrolyte, thereby suppressing dendrite growth. On the other hand, the outer layer, which is rich in crystalline ZnCO_3_, has excellent chemical stability and hydrophobic properties, which not only decreases the water activity between the electrode and electrolyte but also mitigates parasitic reactions such as HER and Zn corrosion. Through this strategy, the authors illustrated that the Zn anode could achieve a high coulombic efficiency of 99.9%. To circumvent the primary challenges of AFZMBs, this bilayer SEI design offers a promising strategy for advanced interphase engineering to overcome the current challenges of Zn anodes, hence paving the way for the development of more efficient and durable AFZMBs.^[^ ^69^ ^]^

In addition to electrolyte additives, in‐situ liquid crystal interphase engineering has recently emerged as an effective strategy to overcome the challenges associated with aqueous batteries.^[^ ^70^ ^]^ Unlike conventional interphase design, this in‐situ liquid crystal interphase uses a soft templating mechanism to facilitate the formation of highly ordered structures within the liquid‐crystalline phases. This highly ordered structure not only promotes rapid ion transport but also reduces the energy barriers for uniform Zn nucleation and deposition.

#### 4.2.2 Hybrid Electrolytes with Advanced Electrolyte Strategies

The use of hybrid electrolytes is also an effective strategy to tackle the critical challenges in AFZMBs, such as stabilizing the reversibility of Zn plating/stripping by developing a more stable electrochemical environment. This is because the addition of highly concentrated salts allows hybrid electrolytes to mitigate parasitic side reactions by reducing the water activity and modulating the solvation structure of Zn^2+^ ions.^[^ ^65^ ^]^ As an example, Ming et al. designed a novel hybrid electrolyte that improves the reversibility and stability of the Zn anode by using the concept of salting‐in effect.^[^ ^71^ ^]^ As shown inFigure 8f, the hybrid electrolyte was prepared by incorporating Zn(OTf)_2_ into propylene carbonate (PC)/water mixtures. The authors observed that PC and water were initially immiscible; however, after the addition of Zn(OTf)_2_, the mixture became completely miscible (Figure 8g). By lowering the water activity interaction and inhibiting side reactions, the Zn^2+^ solvation structure was modified by PC and OTf⁻, which acted as water substitutes in the Zn^2+^ solvation layer. Theoretical simulations revealed that, in a pristine aqueous electrolyte, Zn^2+^ ions are coordinated by six water molecules in the first solvation shell due to water's high dielectric constant.^[^ ^71^ ^]^ On the other hand, when 90% PC is added to the hybrid electrolyte, the solvation layer is dominated by PC and OTf^−^, and only ≈0.5 water molecules coordinate with the Zn^2+^ ion (Figure 8h). Thanks to this unique shift in the solvation structure, a hydrophobic SEI was formed, which acted as a protective layer, that prevented the Zn surface from direct contact with water. This hydrophobic protective layer suppressed side reactions and improved the reversibility of Zn plating/stripping by regulating uniform Zn deposition and dissolution during cycling. As a result, the assembled AFZMBs exhibited a high coulombic efficiency of 99.93% over 500 cycles at a current rate of 1 mA cm^−2^ and achieved a capacity retention of 80% after 275 cycles at 0.5 mA cm^−2^.^[^ ^71^ ^]^ The above approach underlines the future potential of co‐solvent electrolyte engineering in tackling the critical challenges of Zn metal batteries and other anode‐free energy storage systems.

#### 4.2.3 Isosorbide dimethyl ether (IDE)‐Based Electrolytes for stable SEI Formation

**Figure 9:**
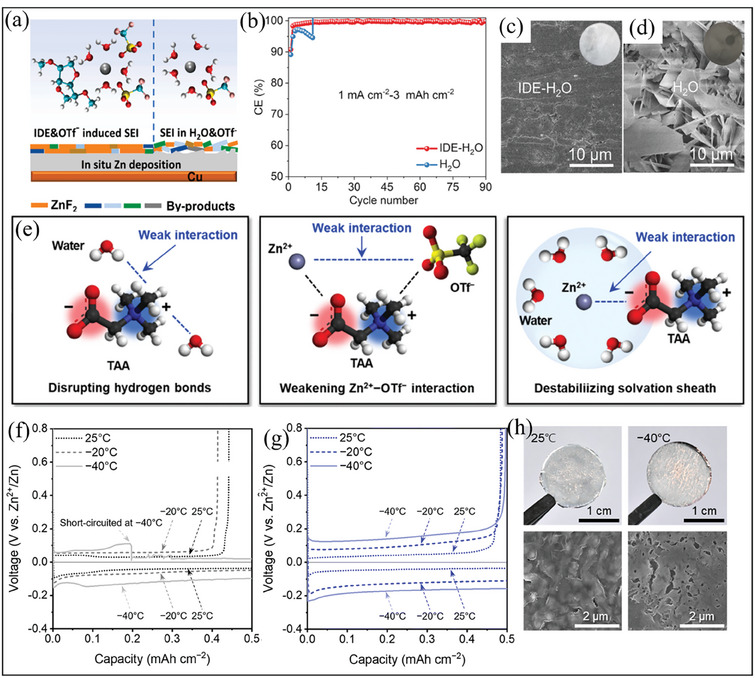
a) Schematic illustrations of the formation of an interfacial layer on Zn anode during Zn plating based on IDE‐H_2_O electrolyte and in pristine aqueous electrolyte. b) Variation of cycle no. with coulombic efficiency. SEM images of Zn metal immersed in c) IDE‐H_2_O electrolyte and d) pristine aqueous electrolyte for 15 days, highlighting the significant differences in surface morphology and corrosion behavior. Reproduced with permission.^[^ ^74^ ^]^ Copyright 2024 Elsevier. e) Schematic representations of soft‐acidic/hard‐basic TAA's Role in stabilizing low‐temperature aqueous electrolytes. Zn plating/stripping curves of Cu substrate with different temperature ranges in f) 1 m Zn(OTf)_2_, and g) the modified ZT‐electrolytes at 0.2 mA cm^−2^ and 0.5 mAh cm^−2^. h) Digital and Post‐mortem SEM images of the Zn plated on a Cu substrate at 25 °C in 1 m Zn(OTf)_2_ (Left) and modified ZT‐electrolytes (Right) at −40 °C, respectively. Reproduced with permission.^[^ ^77^ ^]^ Copyright 2024, The Royal Society of Chemistry.

Another innovative strategy for achieving homogenous Zn deposition and long‐term cycling stability is incorporating organic crowding agents into aqueous electrolytes.^[^ ^72^, ^73^ ^]^ These agents play an important role in modifying the Zn^2+^ solvation structure by reducing the interactions of water activity in aqueous electrolytes, facilitating a stable SEI formation on the Zn surface.^[^ ^72^ ^]^ One notable example is the use of IDE as a crowding agent in AFZMBs.^[^ ^74^ ^]^ Through this electrolyte modification, Wang et al. demonstrated that IDE substitutes the interaction of water activities in the first solvation shell of Zn^2+^ ions, thereby mitigating two primary challenges at the interface‐ i) improving the reversibility of Zn deposition and ii) inhibiting dendrite formation and side reactions. This IDE‐based electrolyte design effectively minimizes water activity, thereby reducing HER and Zn corrosion, which affects the performance of AFZMBs. As illustrated in **Figure** **9**a, a stable and consistent ZnF_2_‐rich SEI is observed with IDE and OTf⁻ (triflate anion), facilitating a more uniform control of Zn deposition on the Cu substrate. In contrast, when Zn is deposited without IDE in an aqueous electrolyte, the SEI is less structured and includes by‐products, resulting in unstable Zn deposition. As a result, the Zn||Cu asymmetric cell with IDE‐H_2_O electrolyte exhibits an outstanding coulombic efficiency of ≈99.80%, highlighting its efficiency in developing high‐areal‐capacity AFZMBs (Figure 9b). The positive impact of this electrolyte modification is further supported by surface chemistry analysis, which demonstrates that Zn deposition with IDE‐H_2_O electrolyte exhibits a smooth and uniform surface, indicating stable Zn growth (Figure 9c). In contrast, Zn deposition in pure aqueous electrolytes is rough and irregular on the surface, signifying uncontrolled Zn growth and poor stability (Figure 9d). Overall, this work illustrates an important concept in electrolyte engineering: shifting the solvation structure and improving the SEI formation are two essential strategies for enhancing the reversibility of Zn deposition in AFZMBs.^[^ ^74^ ^]^ By leveraging these approaches, future electrolyte engineering strategies such as SEI‐forming additives, hybrid electrolytes, or co‐solvent‐based electrolytes can further improve the stability and coulombic efficiency of AFZMBs.

#### 4.2.4 Zwitterion‐Based Electrolytes for Low Temperature

Identifying an ideal aqueous electrolyte with excellent stability and high performance at low temperatures is highly desired for AFZMBs. Unlike traditional aqueous electrolytes, which suffer from low coulombic efficiency and poor cycling stability at sub‐zero temperatures, zwitterion‐based electrolytes emerged as a feasible strategy to enhance the reversibility of Zn deposition while maintaining the ionic conductivity at extremely low temperatures.^[^ ^75^, ^76^ ^]^ For example, to achieve higher coulomic efficiency under extremely low temperatures, Lee et al. recently designed a novel electrolyte by utilizing a soft‐acidic/hard‐basic zwitterion, TAA, (abbreviated as ZT‐electrolyte) into Zn(OTf)_2_ aqueous electrolyte for AFZMBs.^[^ ^77^ ^]^ The results demonstrate that when TAA interacts with the water molecules, it breaks the hydrogen bonding network and reduces the interaction between Zn^2^⁺ ions and OTf⁻ anions, thereby destabilizing the solvation sheath surrounding the Zn^2^⁺ ions, as illustrated inFigure 9e. This modification not only reduces the ion‐pairing interactions but also regulates the Zn^2+^ ion solvation, thereby improving the reversibility of the Zn plating/stripping process.^[^ ^77^ ^]^ As shown in Figure 9f, the assembled Zn||Cu asymmetric cell using ZT‐electrolyte exhibits a stable Zn plating/stripping at −40 °C at a current density of 0.2 mA cm⁻^2^ and areal capacity of 0.5 mAh cm⁻^2^. In contrast, the Zn||Cu asymmetric cell using 1 m Zn(OTf)_2_ electrolyte shows unstable Zn plating/stripping curves at sub‐zero temperatures, ultimately resulting in short‐circuiting at −40 °C due to the accumulation of Zn dendrites (Figure 9g). Moreover, the ZT‐electrolyte facilitates homogeneous and dense Zn deposition without observable dendrite formation at both 25 and −40 °C, as illustrated in Figure 9h. Through this specific electrolyte design, one can improve the reversibility and long‐term stability of aqueous Zn‐based energy storage systems at low‐temperature. Consequently, future electrolyte design strategies should prioritize the optimization of zwitterionic molecules or modify the solvation structure and interfacial stability, including functional co‐solvents or hybrid additives to enhance the low‐temperature performance of AFZMBs.

#### 4.2.5 Comparative Analysis of ZnSO_4_ and Zn(OTf)_2_ Based Aqueous Electrolytes

An essential criterion for increasing the electrochemical performance of AFZMBs is the selection of Zn salts, which profoundly impact Zn^2+^ solvation structure due to their nature of anions, the electrochemical stability window, and electrode stability in aqueous electrolytes.^[^ ^78^ ^]^ Among all the commonly used Zn salts, ZnSO_4_ and Zn(OTf)_2_ are the two most commonly used electrolytes for AFZMBs. It is to be noted here that both of these salts are compatible with AFZMBs, although their optimization strategies are different owing to their inherent characteristics.^[^ ^19^, ^78^ ^]^ For instance, ZnSO_4_‐based electrolytes exhibit high ionic conductivity and good compatibility due to the strong stability of anions; however, they often suffer from poor cycling stability and low coulombic efficiency due to unwanted side reactions, including HER and by‐product formation, which hinders it from developing further.^[^ ^19^ ^]^ However, few strategies have been explored to overcome these challenges, such as using the concept of water‐in‐salt electrolyte or incorporating SEI‐forming additives, i.e., ZnF_2_, to improve the reversibility of Zn plating/stripping.^[^ ^19^ ^]^ On the other hand, the use of Zn(OTf)_2_‐based electrolytes with OTf^−^ anions, as compared to SO_4_^2^‐ anions, can improve the electrochemical performance by decreasing the interaction of water molecules that surround the Zn^2+^ solvation, thereby lowering the solvent effect, and facilitating the release of Zn^2+^ ions more easily from the confined solvation sheath in comparison to ZnSO_4_ electrolyte. Additionally, Zn(OTf)_2_ may suppress the Zn dendrites growth by promoting a stable, anion‐derived SEI, which protects the Zn surface.^[^ ^79^ ^]^ As a result, the Zn(OTf)_2_‐based electrolyte exhibits higher coulombic efficiency and superior cycling stability compared to ZnSO_4_. The optimization strategies for Zn(OTf)_2_ electrolytes include hybrid electrolyte, co‐solvent engineering, or zwitterion incorporation to enhance interfacial stability and reversibility at low temperatures. While both electrolytes have a certain degree of benefits, the current status of electrolyte engineering for AFZMBs is still limited. Future exploration into AFZMBs should focus on solid‐state electrolyte engineering that amalgamates the advantages of both these electrolytes for AFZMBs.

## 5 Challenges Faced by Anode Free Zinc‐Metal Batteries

**Figure 10:**
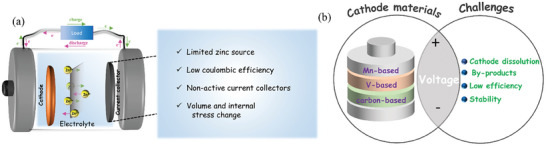
a) Challenges of the current collector, and b) Schematic representation of cathode materials and their respective challenges for AFZMBs.

The above discussion highlights the recent progress in AFZMBs. However, a few key challenges still remain that must be addressed to fully utilize the potential of AFZMBs for practical applications (**Figure** **10**). These include:

1. The low coulombic efficiency and poor cycling stability of AFZMBs mainly arise from the uneven growth of Zn during deposition and stripping processes. In the absence of a pre‐existing anode, Zn is deposited directly onto the current collectors during the charging process. This often leads to irregular and dendritic growth, which reduces the homogeneity of Zn deposition and increases the side reactions. These side reactions can involve the formation of a thick SEI and zinc's reactivity with the electrolyte, which heavily consumes the active Zn and further leads to low coulombic efficiency. Furthermore, during the initial plating/stripping cycle, most AFZMBs lose a significant amount of Zn, reducing their energy density dramatically. In conventional ZMBs, the Zn anode acts as a reservoir to compensate for the Zn loss during cycling. Therefore, improving Zn utilization is essential for the further development of AZMBs.
2. In AFZMBs, the use of nonactive current collectors, such as Cu or titanium (Ti) foils, poses significant challenges, mostly due to the hostless nature of Zn. The absence of a host for Zn promotes dendritic formation on these current collectors. On the other hand, study on the electrochemical performance of cathode electrodes for AFZMBs is limited. Unlike conventional Zn‐based storage, where a metallic Zn anode is the primary source of Zn^2+^ ions, the cathode in AFZMBs is the sole Zn^2+^ source for deposition onto the anode current collectors. This makes selecting cathode material equally important for AFZMBs to achieve high Zn^2+^ storage capacity and long‐term cycling stability.

It is also important here to highlight the current status of cathode materials to overcome the challenges of AFZMBs. At present, only a few cathode materials have been explored for AFZMBs, as shown in **Table** **2** and Figure 10b. These include manganese‐based oxides, i.e., MnO_2_, ZnMnO_2_, vanadium‐based compounds (Zn_3_ V_3_O_8_, Zn_0.5_VO_2_), etc. Although these cathode materials exhibit high theoretical capacities and rapid ion diffusion; however, their practical applications are still limited. This is because most of these materials suffer from material dissolution or structural instability during Zn^2+^ ion storage. Despite these challenges, various optimization strategies have been proposed including pre‐zinc intercalated cathode materials, electrolyte additives, or electrolyte modification to enhance the Zn^2+^ ion storage and cycling stability. Future research and development should focus more on increasing active zinc ions via the use of Zn‐rich cathode or designing advanced cathode material to fully unlock the potential of AFSMBs as a promising energy storage system.

**Table 2:** Summary of electrochemical performance of AFZMBs.

Anode	Cathode	Current Density [mA cm^−2^]	Electrolyte	Asymmetric cells Areal Capacity [mAh cm^−2^]/cycles	Coloumbic efficiency %	AFZMBs Cycling Performance mAh g^−1^/current A g^−1^/Cycles/Capacity Retention	Refs.
C/Cu	MnO_2_	1	3 m Zn(CF_3_SO_3_)_2_+0.1 M Mn(CF_3_SO_3_)_2_	0.5/300	99.6	145/1/80/68.2%	[27]
Cu@AOF	Zn_0.5_VO_2_	10	2 M ZnSO_4_	1/6000	99.95	117/2000/1/60%	[45]
Cu@Cu_3_ Zn alloy	Zn_3_ V_3_O_8_	1	3 M Zn(CF_3_SO_3_)_2_	0.5/300	99.2	60/2/200/80%	[55]
ZA@3D‐nanoCu	Br_2_ MnO_2_	4	1 M ZnSO_4_ and 1 M MnSO_4_	2/1100 h	98.4	450/10/1000/89%	[56]
AgNWA@Cu	ZnMnO_2_	10	2 M ZnSO_4_	40/200	99.8	230/0.5/600/73%	[57]
Sn@NHCF‐Zn	V_2_O_5_	5	2 M ZnSO_4_	5/100	99.7	50/1/2000/100	[58]
Sb/Sb_2_Zn_3_@HI	Carbon felt	20	0.5 M ZnBr_2_ + 0.25 M TPABr	10/550 h	98	10 mAh cm^−2^/800/10 mA cm^−2^	[60]
Cu@Na‐MX@Sn	LiMn_2_O_4_	10	0.5 M ZnBr_2_ + 0.25 M TPABr	20/800 hr	99.52	40.18/100/2/68.9	[61]
PMMA:Zn	ZnMnO_2_	1	3 M Zn(CF_3_SO_3_)_2_	1/2000 h	100	257/1/300/80%	[63]
NC‐Cu@Ti‐0.5	MnO_2_	1	2 M ZnSO_4_	1/400 h	99.2	–	[64]
CuNC@Cu	G/PVP@ZnI_2_	1	5 mM ZnI_2_ + 10 mM I_2_ + 2 M ZnSO_4_	5/1700 h	99.8	1000/1/80%	[67]

## 6 How 3D Printing Technology can Mitigate the Challenges of Anode Free Zinc‐Metal Batteries?

3D printing, also known as additive manufacturing, has shown significant advantages in the field of energy storage devices starting from macroscale to nanoscale applications.^[^ ^80^, ^81^, ^82^, ^83^ ^]^ Unlike conventional manufacturing techniques, 3D printing has distinct advantages, such as designing complex 3D geometries/structures with high surface area and porosity, rapid prototyping, low cost, etc. These unique features make 3D printing an ideal choice for improving AFZMBs performance by enhancing ion and electron transport. Moreover, 3D printing can enhance energy density by integrating porous structures, maximizing available space, and improving overall performance.^[^ ^83^ ^]^ In recent years, various 3D‐printing methods have been reported to fabricate advanced electrodes for electrochemical energy storage devices, with several studies highlighting their potential in aqueous ZMBs.^[^ ^84^, ^85^, ^86^, ^87^, ^88^, ^89^, ^90^ ^]^ In this section, we will not individually discuss each 3D‐printing technique; instead, we will concentrate on the application of 3D printing to tackle the challenges faced by AFZMBs.

### 6.1 3D‐Printed Zn Anodes for Improved Zinc Utilization

**Figure 11:**
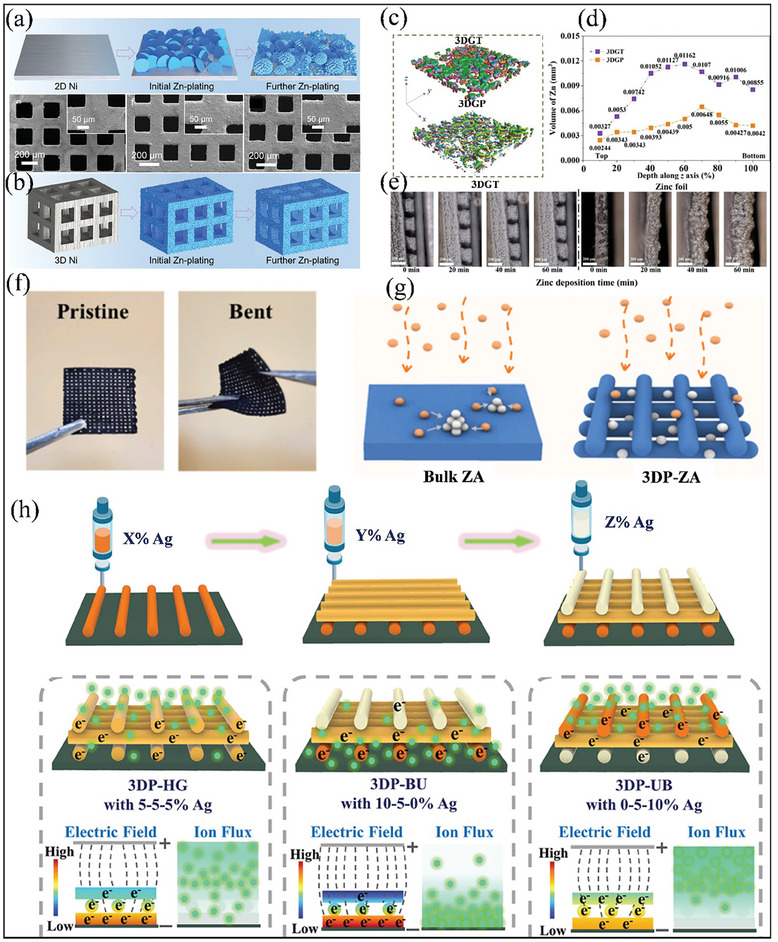
Strategies for 3D printed collectors via 3D printing. Schematic illustrations of Zn plating on a) 2D. b) SEM images and Zn plating distribution on 3D Ni. Reproduced with permission.^[^ ^84^ ^]^ Copyright 2021, Wiley‐VCH GmbH. c) Micro‐CT images of the 3D morphology of Zn plating on 3DGT and 3DGP. d) Volume variations in each layer along the z‐axis. e) In situ optical microscopic images of Zn plating on 3DGPT and bare Zn at a current density of 5 mA cm^−2^. Reproduced with permission.^[^ ^86^ ^]^ Copyright 2022, Elsevier. f) Digital photographs depicting the mechanical flexibility of the 3DP‐ZA anode, shown in both flat (pristine) and bent conditions. g) Schematic representation of Zn plating on bulk‐ZA and 3DA‐ZA anode. Reproduced with permission.^[^ ^94^ ^]^ Copyright 2022, Elsevier, and h) Schematic illustrations of 3D printing scaffolds with layer‐by‐layer Ag % NPs distribution. Reproduced with permission.^[^ ^95^ ^]^ Copyright 2023 Wiley‐VCH GmbH.

A major challenge in AFZMBs is enhancing the coulombic efficiency and cycling stability by improving Zn utilization. To address this, Zhang et al. designed a novel 3D‐printed Zn metal anode with a multichannel lattice structure.^[^ ^84^ ^]^ The constructed 3D Ni‐Zn structures were designed through a combination of 3D printing and electroless plating/electroplating processes. As shown in **Figure** **11**a, the Zn starts to deposit on a 2D Ni surface. However, it can be noticed that Zn deposition becomes less controlled over time, leading to rough and uneven layers, even at a low capacity of 2 mAh cm^−2^, indicating poor stability and higher chances of dendrite formation. On the other hand, the constructed 3D Ni‐Zn with multichannel structures have been shown to uniformly deposit Zn even at a capacity of 2, 5, and 10 mAh cm^−2^ and effectively prevent Zn dendrite growth (Figure 11b). This improvement was due to the unique 3D conductive hosts, which increase the surface area, lower the Zn nucleation overpotential, reduce local current density, and ensure a uniform charge distribution, promoting uniform deposition of metal ions and excellent coulombic efficiency. In order to check the feasibility for practical application, the authors also demonstrated a full cell by assembling the 3D‐printed Ni‐Zn anode and polyaniline‐intercalated vanadium oxide cathodes which exhibit a high energy density of 230 Wh kg^−1^ at 14.2 kW kg^−1^.^[^ ^84^ ^]^

### 6.2 3D‐Printed Host Structures with Dendrite Suppression

Using the 3D printing technique, 3D host structures with interconnected pores and channels can be engineered to mitigate dendritic growth. Jin et al. designed a Zn‐free and dendrite‐free anode based on a 3D‐printed N‐doped hollow carbon nanotube (3DP‐NHC) as a host to obtain favorable microstructure and enhanced rapid reaction kinetics.^[^ ^85^ ^]^ The authors found that 3DP‐NHC not only facilitates rapid diffusion of Zn^2+^ ions but also exhibits uniform charge distribution and low nucleation overpotential. The 3D structure with paratactic columns and surface pores shortens the ion diffusion path, hence resulting in a cycling stability of 420 h at a current rate of 1 mA cm^−2^. Wu and co‐workers further explored this by fabricating two types of 3D printing graphene arrays (3DGs), namely tube arrays (3DGT) and pillar arrays (3DGP), to overcome the limitations of the 2D geometric design of Zn anode.^[^ ^86^ ^]^ The highly ordered 3D printed tube arrays (3DGT) and pillar arrays (3DGP) were fabricated using digital light processing (DLP). The result shows that the highly ordered 3DGT exhibits more Zn deposition than 3DGP. This is due to its internal tunnel architecture and micropores, which provide more space for Zn deposition and electrolyte storage than 3DGP. The selected region inFigure 11c,d was divided into 10 layers along the z‐axis to analyze the Zn deposition in 3D printing graphene arrays (3DGs). Additionally, the distribution of Zn in the top layers was much lower than in the deeper layers, accounting for only 3.72% in 3DGT and 5.67% in 3DGP of the total zinc volume (Figure 11d). On the contrary, in‐situ optical characterization was performed to visualize the uniformity of Zn deposition. As illustrated in Figure 11e, the distribution of Zn deposition on 3DGT preserved the separators intact at the interface, preventing the formation of dendrites. On the other hand, the surface of Zn foil exhibited severe surface evolution and dendritic formation within several thicknesses during the deposition.^[^ ^86^ ^]^ Thus, these observation indicates that the highly ordered 3DGT can efficiently regulate the Zn deposition and suppress the dendrite formation.

### 6.3 Engineered 3D‐Printed with High Mechanical Flexibility and Stability

Recently, considerable efforts have been focused on using 3D printing to design flexible Zn anodes with outstanding mechanical stability based on lightweight substrates for flexible energy storage devices.^[^ ^87^, ^88^, ^89^, ^90^, ^91^ ^]^ The limitations of the conventional substrates are that they suffer from mechanical mismatch with Zn deposits, which are typically rigid, and this can lead to microcrack formations leading to dendritic growth after several deformation cycles. While Zn powder can create a flexible Zn film, the resulting structure can still suffer from uneven distribution and stress concentration, promoting dendrite formation and large volume changes during Zn plating/stripping.^[^ ^92^, ^93^ ^]^ Therefore, uniform Zn deposition and negligible inner stress are highly required to meet the demand for excellent mechanical flexibility. Zhang et al. fabricated a unique 3D‐printed, silver‐anchored, hierarchical porous flexible Zn anode (3DP‐ZA) via 3D printing for applications in flexible ZMBs.^[^ ^94^ ^]^ The 3DP‐ZA comprises an interconnected carbon matrix, chemically linked with uniformly distributed Zn and zincophilic Ag particles, ensuring great structural stability and flexibility (Figure 11f). In addition, the 3DP‐ZA anode provides a highly interconnected porous framework that facilitates uniform Zn deposition throughout the structure and prevents dendrite formation, as illustrated by the even distribution of Zn^2^⁺ ions compared to the dendrites observed in the Bulk‐ZA (Figure 11g). This unique structure, composed of zincophilic Ag particles and a carbon matrix, allows for better stress release and maintains structural stability during several cycles, making it a superior choice over conventional bulk anodes.^[^ ^94^ ^]^ Following this work, the same group also reported a 3D‐printed anode with a unique dual‐gradient design that regulates both electron and ion transport among the electrode, resulting in more uniform Zn plating and stripping during charge/discharge cycles.^[^ ^95^ ^]^ The procedure for the fabrication of the three‐layer 3D‐printed structure with varying concentrations of Ag % is shown in Figure 11h. By regulating Ag % concentration gradients inside each layer, the authors could optimize the equilibrium between electron transport, essential for effective charging and discharging, and ion mobility, crucial for Zn deposition. The bottom‐up gradient (3DP‐BU) as shown in Figure 11h, where the Ag concentrations of the bottom, middle, and top layers are 10, 5, and 0 wt.%, appears to provide an ideal equilibrium, mitigating dendrite formation and prolonging battery lifespan in comparison to 3DP‐HG (5–5–5 wt.%) and 3DP‐UB (0–5–10 wt.%). The capacity to manipulate electron and ion behavior via 3D printing represents a substantial advancement for next‐generation Zn batteries.^[^ ^95^ ^]^

### 6.4 Engineered High‐Performance 3D‐Printed Cathodes

**Figure 12:**
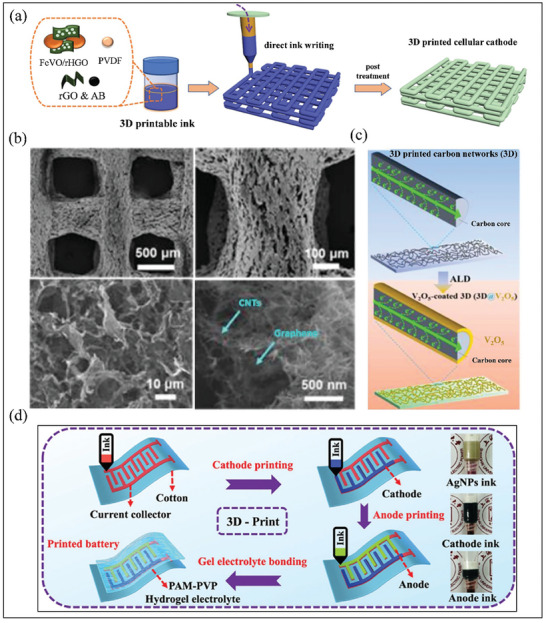
Strategies for 3D printed cathode electrodes. a) Schematic illustrations for the preparation of 3D printed cathode electrodes for ZMBs. Reproduced with permission.^[^ ^87^ ^]^ Copyright 2021, Wiley‐VCH. b) SEM images of the 3D‐CM surfaces at different magnifications. Reproduced with permission.^[^ ^96^ ^]^ Copyright 2023, Wiley‐VCH. c) Schematic representation of the fabrication process for V_2_O_5_‐coated 3D‐printed carbon electrode. Reprinted with permission.^[^ ^97^ ^]^ Copyright 2021, Wiley‐VCH, and d) Schematic representation of the 3D printing stages, including embedded optical images depicting the collector, cathode, and anode inks used to fabricate 3D‐printed zinc‐ion micro‐batteries. Reproduced with permission.^[^ ^88^ ^]^ Copyright 2023, Wiley‐VCH.

3D‐printing technology is also being applied to design advanced cathodes for aqueous ZMBs. Cao et al. designed a 3D‐printed cathode using ink composed of Fe_5_V_15_O_39_(OH)_9_·9H_2_O (FeVO) nanosheets and reduced holey graphene oxide (rHGO), as illustrated in **Figure** **12**a. The 3D‐printed FeVO/rHGO cathodes achieved a high specific capacity of 344.8 mAh g^−1^ at 0.1 A g^−1^ and demonstrated exceptional cycling stability over 650 cycles at 2 A g^−1^.^[^ ^87^ ^]^ These 3D‐printed cathodes are composed of periodic macroporous channels and hierarchical porous structures, which facilitate electrolyte ion infiltration and significantly enhance the fast transport of electrons and ions. To promote the practical application of aqueous ZMBs, Wu et al. also integrated MnO_2_ onto 3D‐printed carbon microlattices (3DP CMs).^[^ ^96^ ^]^ They utilized graphene oxide (GO) and carbon nanotube (CNT) ink to directly print the lattice, followed by high‐temperature annealing to boost conductivity and generate surface defects. The structural benefits of 3DP CMs greatly enhance the electrochemical deposition of MnO_2_, leading to a significantly lower overpotential compared to conventional current collectors like Ti mesh and carbon cloth. A top view of the 3DP CMs at various magnifications is shown in Figure 12b. The designed MnO_2_ cathode enabled the ZMBs to achieve an energy density of 226.2 Wh kg⁻¹ and 6.4 mWh cm⁻^2^ in terms of gravimetric and areal metrics, respectively. On the other hand, Gao and co‐workers reported another study on a vanadium‐based 3D‐printed cathode for aqueous ZMBs, where they designed a hierarchical core‐shell structured cathode (3D@V_2_O_5_) by integrating fused deposition modeling (FDM) 3D printing with atomic layer deposition (ALD).^[^ ^97^ ^]^ In this study, a porous carbon current collector was printed using FDM, and the active material V_2_O_5_ was deposited onto the porous carbon network through atomic layer deposition (Figure 12c). The 3D‐printed porous carbon network offers a highly entangled, electron‐conductive framework along with interconnected pathways of Zn^2^⁺ ion diffusion, imparting the 3D@V_2_O_5_ cathode with remarkable specific capacity and good rate capability.

Recently, Wang and co‐workers reported a ground‐breaking study on 3D‐printed aqueous ZMBs, in which they developed a printed zinc‐ion micro‐battery (PZMB) using 3D printing technology.^[^ ^88^ ^]^ For the printing process, polyvinylpyrrolidone‐induced ammonium vanadate (P‐NVO) nanobelts were employed as the cathode, while zinc chlorate hexahydrate [Zn(ClO_4_)_2_·6H_2_O] served as the electrolyte salt to create a polyacrylamide (PAM)‐polyvinylpyrrolidone (PVP) double‐network hydrogel electrolyte. The fabricated PZMBs exhibited a high areal capacity of 4.02 mAh cm⁻^2^ at 0.5 mA cm⁻^2^ and impressive mechanical flexibility. The structure of the 3D‐printed zinc‐ion micro‐battery is illustrated in Figure 12d. Mattevi and co‐workers designed a 3D‐printed VS_2_ cathode using the direct ink writing 3D‐printing method to create full zinc‐ion cells.^[^ ^98^ ^]^ The full cell, assembled with 3D‐printed VS_2_ cathodes and carbon/Zn foil anodes, demonstrated a capacity of ≈1.98 mAh cm^−2^ at an operating voltage of 1.5 V, showing good reversibility over 150 cycles with stable charge and discharge curves.

Based on the above discussion, we propose that the integration of 3D printing technology can address the key challenges in AFZMBs, from ensuring uniform Zn deposition to mitigating the dendritic growth of Zn and boosting mechanical flexibility and ion transport. The design and fabrication of Zn‐rich cathodes could also offer more efficient control over material architecture, which conventional techniques could not achieve. Additionally, 3D printing enables the fabrication of intricately structured that offer a more uniform charge distribution, which is crucial for improving the cycling stability and overall battery lifespan.

## 7 Conclusion and Future Perspectives

The development of AFZMBs represents a significant breakthrough in addressing the challenges of conventional ZMBs. By eliminating the need for pre‐formed Zn anode, AFZMBs enhance energy density, reduce weight, and improve high safety. This cutting‐edge design addresses key challenges like dendrite formation and corrosion, which typically undermine the stability and performance of conventional ZMBs. Nonetheless, several challenges remain that must be addressed before they can achieve widespread commercial viability. The limitation of low coulombic efficiency and heterogeneous Zn deposition are among the most significant limitations encountered by AFZMBs. In this review, we have discussed the various strategies, including current collector modification and electrolyte engineering, in an effort to mitigate these issues.

However, additional research is required to completely overcome the instability caused by Zn dendrites and enhance the overall coulombic efficiency and cycling stability of AFZMBs. In this context, 3D printing technology presents a promising opportunity for developing next‐generation AFZMBs. The capacity to create porous current collectors and cathodes with intricate structures allows for more precise control over Zn deposition, thereby reducing the risk of dendrites and enhancing uniformity. Furthermore, the performance of batteries can be considerably improved by using 3D‐printed designs, which can significantly improve ion and electron transport. The customization potential of 3D printing allows researchers to tailor battery components to meet specific requirements, thereby optimizing material utilization and generating more lightweight and efficient designs. Consequently, incorporating 3D printing into AFZMB development could greatly expedite their practical use in next‐generation energy storage systems. Based on these, several key prospects for the further development of AFZMBs are highlighted below:

1. Electrolyte Optimization: While aqueous‐based electrolytes have numerous advantages in AFZMBs, flexible AFZMBs remain a completely unexplored area. It is crucial to concentrate on developing and investigating gel or solid‐state electrolytes for applications in wearable devices. These electrolytes not only promote flexibility and facilitate the design of more diverse batteries but also improve safety by mitigating problems such as dendrite growth and electrolyte leakage, resulting in more stable and better battery performance.
2. Designing current collectors and advanced cathodes via 3D printing technique: 3D printing technology is a viable solution to mitigate the challenges faced by AFZMBs by allowing the design of intricately structured and porous current collectors, which can promote homogeneous Zn plating and stripping. Additionally, it can facilitate the incorporation of protective coatings and specialized material treatments, reducing corrosion and hydrogen evolution, hence enhancing the durability and stability of AFZMBs. For instance, conventionalCu substrates, typically used as current collectors, have difficulties stemming from inadequate adhesion with Zn and restricted surface area, resulting in uneven Zn deposition and low coulombic efficiency in anode‐free cells. To mitigate this, using 3D conductive substrates with increased surface areas and porosity can reduce the local current density, stabilizing Zn plating. This method has demonstrated efficacy in cells containing excess Zn and other monovalent batteries. Besides, investigations on cathode electrodes for AFZMBs are still limited. However, the cathode electrode is crucial in these batteries; hence, optimizing the cathode using the 3D printing technique is essential to unlock the potential of AFZMBs for practical applications fully. By utilizing the 3D printing technique, the cathode electrode for AFZMBs can be designed with precise control over its architecture, enabling the creation of porous and highly interconnected structures that enhance both electron and ion transport. This level of control also allows for the optimization of material distribution, increasing the surface area for zinc‐ion interactions and improving the battery performance. Besides, the customizable nature of 3D printing can further help in designing zinc‐rich cathodes that promote uniform Zn deposition during cycling, which is crucial for minimizing the dendrite growth common issue in ZMBs.
3. Advanced spectroscopic techniques and computational methods: The future development of AFZMBs should utilize sophisticated spectroscopic in situ techniques (in situ TEM, XRD, Raman, operando, etc.) to unveil the behavior of the electrode‐electrolyte interface, and combined with computational methodologies like DFT and molecular dynamics simulations to understand the in‐depth mechanism.
4. Optimize separators: In AFZMBs, separators are the least discussed. However, inhibiting direct contact between the cathode and the Zn deposits is crucial, thereby reducing the risk of short circuits. Therefore, optimizing the separators by improving the pore size, mechanical strength, etc., is of utmost importance in improving the battery lifespan.
5. Economic scalability and safety of the battery: In AFZMBs, special focus should be given to augmenting environmental stability and safety to improve their practical uses. In addition to battery safety for large‐scale systems, advancements in material design, intelligent battery monitoring systems, real‐time failure detection, life cycle prediction, and data‐driven battery management systems can greatly enhance performance and reliability. These technologies will increase efficiency and ensure greater safety, supporting the long‐term success of battery solutions.

## Conflict of Interest

The authors declare no conflict of interest.
